# Chronic IL-1 Exposure Attenuates RELA- and STAT3-Dependent Synergistic Cytokine Signaling in Prostate Cancer Cell Lines

**DOI:** 10.3390/cancers17233778

**Published:** 2025-11-26

**Authors:** Stephanie Akemi Yamauchi, Haley Dahl-Wilkie, Mohamed Hussien Mohamed Zaky, Vivian Liu, Adora Onuogu, Ahmed Abdi, Shreya Billa, Rahael Javaid, Sheza Siddiqui, Chisom Mbah, Olaoluwapo Bankole, Sarah Wells, Sydney Greene, Rafah Falah, Nikki Ayanna Delk

**Affiliations:** Biological Sciences Department, The University of Texas at Dallas, Richardson, TX 75080, USA; stephanie.yamauchi@utdallas.edu (S.A.Y.); hcd160130@utdallas.edu (H.D.-W.); mohamed.zaky@utdallas.edu (M.H.M.Z.); liuxvivian@gmail.com (V.L.); adoraonuogu@gmail.com (A.O.); aja180001@utdallas.edu (A.A.); shreyabilla8@gmail.com (S.B.); rahael.javaid@utdallas.edu (R.J.); sheza.siddiqui@utdallas.edu (S.S.); chisom.mbah@utdallas.edu (C.M.); olaoluwapo.bankole@utdallas.edu (O.B.); sarah.wells@utdallas.edu (S.W.); greene5@wharton.upenn.edu (S.G.); rfalah@utdallas.edu (R.F.)

**Keywords:** prostate cancer, inflammation, cytokine, cell proliferation

## Abstract

Background/Objectives: The milieu of inflammatory cytokines present in the prostate cancer (PCa) tumor microenvironment exerts various effects on cancer progression. Chronic exposure to the inflammatory cytokine interleukin-1 (IL-1) has been shown to impact signaling via the RELA/NF-kB pathway; however, the effects of chronic inflammation on the integration of different inflammatory signaling pathways, such as the interleukin-6 (IL-6)/STAT3 axis, requires further exploration. Methods: We generated in vitro subline models by exposing the C4-2 and LNCaP PCa cell lines to either IL-1α or IL-1β for several months. We then treated the resulting sublines with acute IL-1 alone, IL-6 alone, or IL-1/IL-6 in combination and assessed for sensitivity to cytokine signaling. We observed changes in proliferation and quantified using Ki-67 immunostaining. Cell proliferation was assessed after siRNA silencing *RELA* or *STAT3*. Results: IL-1/IL-6 signaling in combination enhanced the signaling effects of either cytokine alone, particularly cytostasis. While the chronic IL-1 sublines maintained sensitivity to acute IL-6 signaling, they lost sensitivity to acute IL-1 signaling and did not show the enhanced IL-1/IL-6 cytostatic response. Inhibition of *RELA* and *STAT3* rescued cytostasis after IL-1/IL-6 treatment in parental PCa cell lines, but only *STAT3* inhibition rescued proliferation in the chronic IL-1 sublines. Conclusions: Our work shows that IL-1/RELA and IL-6/STAT3 work in parallel to synergistically induce cytostasis. However, chronic IL-1 exposure selects for cells that attenuate IL-1/RELA signaling, subsequently attenuating IL-1/IL-6 synergy.

## Simple Summary

Chronic inflammation promotes prostate cancer (PCa) progression. In this study, we examined the effect of chronic exposure to interleukin-1 (IL-1) inflammatory cytokine on the ability of two PCa cell lines to integrate signals from another inflammatory cytokine, interleukin-6 (IL-6). We found that chronic IL-1 treatment attenuates PCa cells’ synergistic response to IL-1 and IL-6 in combination, particularly with regard to cell proliferation. Our research shows that chronic inflammation can alter crosstalk among different inflammatory signaling pathways. This emphasizes the importance of comprehending the integration of signals in the dynamic inflammatory tumor microenvironment to better understand tumor development and for therapeutic intervention.

## 1. Introduction

Inflammation is a hallmark of cancer [1] and has been shown to influence various aspects of cancer cell activity, including cell proliferation, metastasis, hormone dependence, and treatment resistance [2,3]. The prostate cancer (PCa) tumor microenvironment contains elevated levels of many different pro-tumorigenic inflammatory cytokines, including interleukin-1 (IL-1) [4]. IL-1′s pro-tumorigenic functions include promoting tumor angiogenesis [5] and metastasis [6,7,8], and elevated IL-1 accumulation in PCa patient serum and tumor tissue is associated with disease progression and poor prognosis [6,9].

Chronic inflammation (i.e., prolonged and unresolved inflammatory response) has been linked to increased risk of cancer progression, including PCa progression [10,11]. For example, we and others have previously shown that chronic IL-1 exposure selects for PCa cells that develop androgen receptor independence and treatment resistance [12,13]. In addition, we found that chronic IL-1 exposure selects for PCa cells that lose or evolve reduced sensitivity to IL-1, and in doing so, leads to the loss of sensitivity to tumor necrosis factor alpha (TNFα) [12]. Thus, chronic IL-1 exposure alters PCa cell response to other exogenous inflammatory stimuli. Like IL-1, TNFα signals via the canonical RELA/NF-kB pathway [3], suggesting that chronic IL-1 specifically disrupts components of the RELA/NF-kB pathway. Herein, we investigate the effect of chronic IL-1 exposure on interleukin-6 (IL-6) response. IL-6 signals through JAK/STAT [14] and has been shown to crosstalk with IL-1/NF-kB signaling in PCa cells [15]. Both IL-1 [16,17,18] and IL-6 [17,19,20] can induce the neuroendocrine differentiation phenotype in PCa cell lines and promote castration resistance. Furthermore, elevated IL-6 in PCa serum and tumor tissue is associated with disease progression and poor prognosis [4,21]. Therefore, the IL-1/IL-6 axis is functionally and clinically relevant in PCa.

To determine the effect of chronic IL-1 exposure on the IL-1/IL-6 axis, we exposed LNCaP [12] and C4-2 cells to IL-1 for several months to generate chronic IL-1 sublines. We then tested parental control and subline cell sensitivity to IL-1 or IL-6 alone or in combination. We found that IL-1/IL-6 signaling in combination synergistically enhances RELA- and STAT3-dependent cytostasis over either cytokine alone in the PCa parental cell lines. The chronic IL-1 subline cells, however, evolve IL-1 insensitivity resulting in attenuated IL-1/IL-6 signaling. Together, our results support that chronic inflammation in the tumor microenvironment alters how cancer cells integrate varying signaling pathways.

## 2. Materials and Methods

### 2.1. Cell Culture

C4-2 (ATCC, Manassas, VA, USA; CRL-3314) and LNCaP (ATCC, Manassas, VA, USA; CRL-1740) prostate cancer (PCa) cell lines were maintained in a 37 °C, 5.0% (*v*/*v*) CO_2_ growth chamber, cultured in Dulbecco Modified Eagle Medium (DMEM (Gibco/Thermo Scientific; Grand Island, NY, USA; 1185-092) supplemented with 10% (*v*/*v*) fetal bovine essence (FB Essence (FBE); Seradigm, Radnor, PA, USA; 3100-500), 0.4 mM L-glutamine (L-glut; Gibco/Invitrogen, Paisley, PA, USA; 25030-081), and 10 U/mL penicillin G sodium and 10 mg/mL streptomycin sulfate (pen-strep; Gibco/ Invitrogen, Grand Island, NY, USA; 15140-122).

### 2.2. Chronic IL-1 Subline Generation and Maintenance

To generate chronic IL-1 sublines, C4-2 cells were cultured in DMEM/10% FB Essence (FBE) containing 0.5 ng/mL of IL-1α (Gold Bio, St. Louis, MO, USA; 1110-01A-10) or IL-1β (Gold Bio, St. Louis, MO, USA; 1110-01B-10) for 6 months, the time it takes to establish proliferative, IL-1-insensitive colonies. We refreshed IL-1 treatment every 3–5 days to maintain IL-1 potency. Following 6 months chronic IL-1 exposure, cells were moved to and maintained in normal growth medium without supplemental IL-1 to establish stable sublines. IL-1 concentration and chronic exposure time course were based on previous publications [12,13]. We generated two sets of independent C4-2 sublines, which we refer to as C4-2 chronic IL-1α sublines 1 and 2 (C42as1, C42as2) and C4-2 chronic IL-1β sublines 1 and 2 (C42bs1, C42bs2). During subline generation, C4-2 parental cells 1 and 2 (C4-2 Par1, C4-2 Par2) were cultured in vehicle control (1× phosphate buffer saline, PBS) (Corning, Manassas, VA, USA; 21-040-CM) alongside the sublines. The generation of the LNCaP parental (LNCaP-1) and LNCaP chronic IL-1 sublines (LNas1, LNbs1) has been previously described [12]. Cell line authentication for the C4-2 and LNCaP parental and sublines was performed by STR profiling by the DNA Genotyping Core, UTSW Medical Center. We hereby confirm that none of the cell lines used required any ethics approval for their use.

### 2.3. Cell Treatments

#### 2.3.1. Cytokines

Human recombinant IL-1α (GoldBio, St. Louis, MO, USA; 1110-01A-100), IL-1β (GoldBio, St. Louis, MO, USA; 1110-01B-100), or IL-6 (R&D Systems, Minneapolis, MN, USA; 206-IL-010) was resuspended in 0.1% bovine serum albumin (BSA) (Thermo Fisher Scientific, Fair Lawn, NJ, USA; BP 1600-1) in 1× phosphate buffered saline (PBS; Corning, Manassas, VA, USA; 21-040-CM). Cells were treated with vehicle control (0.1% BSA in 1× PBS), IL-1α, IL-1β, or IL-6 added to DMEM/10% FBE growth medium.

#### 2.3.2. Gene Silencing (siRNA)

The following siRNA concentrations were used: 70 nM, 90 nM, or 140 nM non-targeting siRNA (Dharmacon, Lafayette, CO, USA; D-001206-14-20); 70 nM *RELA* siRNA (Dharmacon, Lafayette, CO, USA; M-003533-02-0005); and 70 nM or 90 nM *STAT3* siRNA (Dharmacon, Lafayette, CO, USA; M-003544-02-0005). Cells were transfected with siRNA for 1 day using siTran 1.0 transfection reagent (Origene, Rockville, MD, USA; TT300003) or TransIT-X2 Dynamic Delivery System (Mirius, Madison, WI, USA; MIR 6003) in DMEM/10% FBE growth medium. The next day, the medium was diluted or replaced with fresh DMEM/10% FBE, plus IL-1, IL-6, or the vehicle control, for 3 days.

### 2.4. RNA Analysis

#### 2.4.1. RNA Isolation and Reverse Transcription Quantitative PCR (RT-qPCR)

RNA isolation and RT-qPCR were performed as previously described [12].

#### 2.4.2. Primer Sequences

5′-3′: *Beta actin* (*β-actin*), forward GATGAGATTGGCATGGCTTT, reverse CACCTTCACCGGTCCAGTTT; *Mitochondrial Superoxide Dismutase 2* (*SOD2*), forward GGCCTACGTGAACAACCTGA, reverse GTTCTCCACCACCGTTAGGG; *Lipocalin-2* (*LCN2*), forward TCACCCTCTACGGGAGAACC, reverse GGGACAGGGAAGACGATGTG; *RELA*, forward TGAACCAGGGCATACCTGTG, reverse CCCCTGTCACTAGGCGAGTT.

### 2.5. Protein Analysis

#### 2.5.1. Western Blot

Protein isolation and Western blot were performed as previous described [12].

#### 2.5.2. Antibodies

Primary antibodies: SOD2 (Cell Signaling Technology, Danvers, MA, USA; 1314S), LCN2 (Cell Signaling Technology, Danvers, MA, USA; 44058S), STAT3 (Cell Signaling Technology, Danvers, MA, USA; 9139S), pSTAT3 (Cell Signaling Technology, Danvers, MA, USA; 4113S), p65 (Cell Signaling Technology, Danvers, MA, USA; 8242S), and β-actin (Santa Cruz, Santa Cruz, CA, USA; sc-69879). Secondary antibodies: sheep anti-mouse (Jackson ImmunoResearch Laboratories, Grove, PA, USA; 515-035-062), goat anti-rabbit (Abnova, Walnut, CA, USA; PAB10822).

### 2.6. Immunofluorescence

#### 2.6.1. Staining

Cells were plated into 48 well plates (Nunclon, Roskilde, Denmark; 150687). Cells were fixed and permeabilized with 100% methanol at −20 °C for at least 30 min. Antibodies were diluted in 2.5% BSA in 1× PBS. Nuclei were stained with DAPI (10236276001; Roche Diagnostics, North Ryde, NSW, Australia).

#### 2.6.2. Antibodies

Primary antibodies: Ki-67 (Millipore, Darmstadt, Germany, MAB4190). Secondary antibodies: goat anti-mouse (Invitrogen, Rockford, IL, USA, A32723).

#### 2.6.3. Cell Counts

The ratio of proliferating cells was determined by counting Ki-67-positive cells and cells stained with DAPI using the Cytation3 Cell Imaging Multi-Mode Reader (BioTek, Winooski, VT, USA). *n* = 9362–137,330 cells were counted for a given biological replicate.

### 2.7. Statistical Analysis

Statistical significance was determined using an unpaired Student’s *t*-test calculated using Microsoft Excel. *p*-values of ≤0.05 were considered to be statistically significant and denoted by asterisks (* *p* ≤ 0.05; ** *p* ≤ 0.05; *** *p* ≤ 0.005). “NS” indicates no statistical significance. Error bars indicate ±standard deviation (SD); *n* ≥ 3 biological replicates. Experiments were repeated at least three times and representative data are shown.

## 3. Results

### 3.1. Chronic IL-1 Exposure Selects for Cells That Lose or Attenuate Sensitivity to IL-1

**Figure 1:**
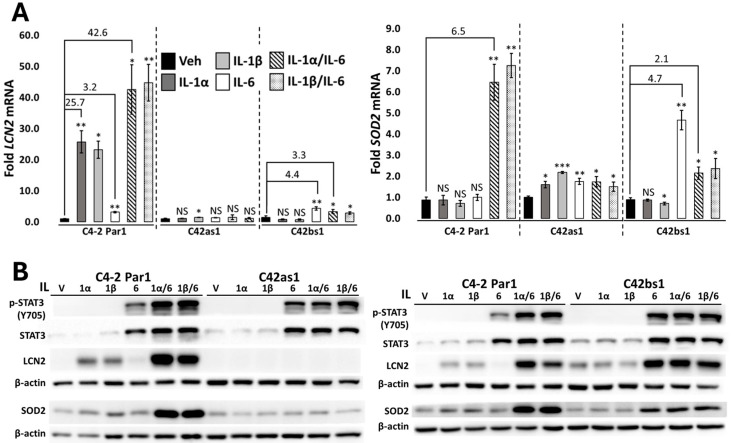
Chronic IL-1 exposure attenuates IL-1 and IL-1/IL-6 intracellular signaling. C4-2 Par1 parental cells and the chronic IL-1 sublines C42as1 and C42bs1 were treated with vehicle control, 25 ng/mL IL-1α, 25 ng/mL IL-1β, or 100 ng/mL IL-6 alone or in combination acutely for 4 days. (**A**) RNA and (**B**) protein were collected and analyzed via RT-qPCR and Western blot, respectively. IL-1 sensitivity was assessed using the canonical IL-1-induced genes *LCN2* and *SOD2*. IL-6 sensitivity was assessed using phosphorylated STAT3 (p-STAT3) accumulation. Acute IL-1 exposure induced LCN2 mRNA and protein levels in C4-2 Par1 cells, but comparatively, LCN2 induction was not detectable in the C42as1 or C42bs1 chronic IL-1 subline cells. IL-1 did not induce SOD2 mRNA or protein in parental or subline cells. IL-6 induced both total STAT3 and p-STAT3 accumulation in the parental and subline cells and induced both SOD2 and LCN2 in the C42bs1 subline only. Finally, IL-1/IL-6 combination induced enhanced LCN2 or SOD2 levels in C4-2 Par1 cells but not in the C42as1 or C42bs1 chronic IL-1 subline cells. Taken together, chronic IL-1 exposure attenuates IL-1 and IL-1/IL-6 intracellular signaling. *n* = 3 biological replicates; error bars = +/−STDEV; *p*-value = * ≤0.05, ** ≤0.005, *** ≤0.0005. Fold mRNA levels were normalized within the cell line to the vehicle control, and the numerical values indicate fold change. Original western blots are presented in File S1.

Previously, we generated a novel chronic inflammation subline model by exposing LNCaP PCa cells to either IL-1α or IL-1β for 3-4 months to generate a LNCaP IL-1α subline (LNas1) and LNCaP IL-1β subline (LNbs1). As compared with the LNCaP parental cell line cultured alongside in vehicle control (LNCaP-1), both LNas1 and LNbs1 developed insensitivity to acute IL-1 treatment [12]. To determine whether the PCa cell line C4-2 would evolve similar insensitivity to IL-1 post chronic exposure, two sets of C4-2 chronic IL-1 sublines were generated by culturing cells in 0.5 ng/mL of IL-1α, IL-1β, or vehicle control for 6 months to generate, respectively, two C4-2 IL-1α sublines (C42as1 and C42as2), two C4-2 IL-1β sublines (C42bs1 and C42bs2), and two C4-2 parental control cell lines (C4-2 Par1 and C4-2 Par2). To assess cell response to acute IL-1, we treated C4-2 parental and chronic IL-1 subline cells with 25 ng/mL of IL-1α or IL-1β for 4 days. The IL-1 target gene, Lipocalin-2 (*LCN2*), was analyzed for RNA expression via RT-qPCR and for protein accumulation by Western blot. Acute IL-1 treatment induced *LCN2* expression in C4-2 Par cells but not in the C4-2 chronic IL-1 sublines (Figure 1A and Figure S1A). Similar results were observed by Western blot (Figure 1B and Figure S1B). Thus, as previously observed for LNCaP cells ([12], Figure S1), both chronic IL-1α and chronic IL-1β exposure select for C4-2 PCa cells that lose or attenuate sensitivity to acute, exogenous IL-1 treatment. Also, as previously observed for LNCaP cells [12], *IL-1R1* receptor levels remain comparable in C4-2 parental and subline cells (Figure S1C). Our results show that chronic IL-1 exposure elicits a conserved IL-1 insensitivity response in both PCa cell line backgrounds that likely is not due to reduced receptor levels.

### 3.2. Chronic IL-1-Exposed Cells Can Still Activate IL-6/JAK/STAT Signaling

One consequence of chronic IL-1 signaling on PCa cells is attenuated or lost response to other NF-kB-mediated inflammatory factors besides IL-1 [12]. Another clinically relevant inflammatory cytokine present in the PCa tumor environment is IL-6 [4,14,21]. IL-6 has been linked to the promotion of PCa bone metastasis, neuroendocrine differentiation, castration resistance, and cell proliferation [8,14,20]. IL-6 binds the cell surface IL-6 receptor to induce JAK kinase phosphorylation and transactivation of STAT3 [14]. To determine if chronic IL-1 exposure can alter cell response to NF-kB-independent inflammatory cytokines, we assessed molecular response to IL-6. To assess the functionality of IL-6 signaling in PCa cell lines after chronic IL-1 treatment, we treated the C4-2 and LNCaP chronic IL-1 sublines acutely with 100 ng/mL of IL-6 for 4 days. We assessed total STAT3 and phosphorylated STAT3 (p-STAT3, Y705) protein accumulation via Western blot. Our acute IL-6 treatment was sufficient to induce both total STAT3 accumulation and STAT3 phosphorylation in C4-2 and LNCaP parental cells and in the chronic IL-1 subline cells (Figure 1B and Figure S1B). Thus, chronic IL-1 exposure does not inhibit canonical IL-6/JAK/STAT signal activation.

### 3.3. IL-1 and IL-6 Axes Crosstalk to Enhance Intracellular Signaling

The PCa tumor microenvironment hosts a variety of inflammatory cytokines, where cancer cells are integrating multiple different signals from multiple different cytokines to influence cancer cell behavior [10,11]. While IL-1 signals through the RELA/NF-kB pathway [3] and IL-6 through the JAK/STAT pathway [14], synergistic crosstalk has also been observed between these cytokine pathways in PCa cell lines [15]. In kind, we investigated synergistic IL-1/IL-6 crosstalk in C4-2 and LNCaP cells. We treated C4-2 and LNCaP parental cells with 25 ng/mL of IL-1α or IL-1β in combination with 100 ng/mL of IL-6 for 4 days. We assessed IL-1 response by RT-qPCR or Western blot for the canonical IL-1-induced genes *LCN2* and *Superoxide Dismutase 2* (*SOD2*) and assessed the IL-6 response by Western blot for STAT3 and phosphorylated STAT3 protein accumulation. The IL-1/IL-6 combination treatment induced *LCN2* and/or *SOD2* mRNA and protein levels to a greater extent than IL-1 or IL-6 alone in C4-2 and LNCaP parental cell lines (Figure 1 and Figure S1). While inconsistent for both sets of C4-2 parental cells, we found that STAT3 phosphorylation was higher in the LNCaP-1 parental cells treated with the IL-1/IL-6 combination than with either cytokine alone (Figure 1B and Figure S1B). Taken together, our data shows that IL-1 and IL-6 intracellular pathways crosstalk and that this crosstalk can enhance intracellular signaling.

### 3.4. IL-1 and IL-6 Crosstalk Synergistically Induces Cytostasis

**Figure 2:**
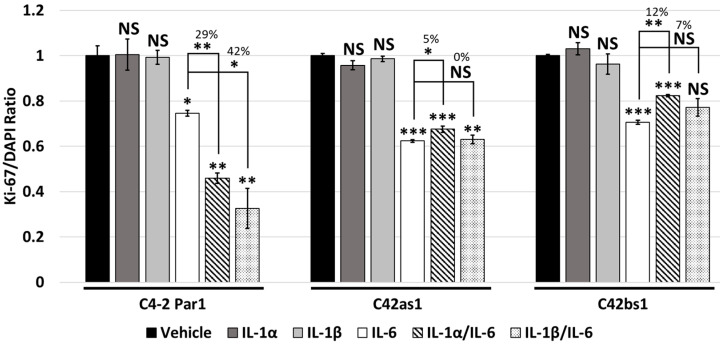
Chronic IL-1 exposure attenuates IL-1/IL-6-induced cytostasis. C4-2 Par1 parental and chronic IL-1 subline cells C42as1 and C42bs1 were treated with vehicle control, 25 ng/mL IL-1α, 25 ng/mL IL-1β, or 100 ng/mL IL-6 alone or in combination for 4 days. Following treatment, cells were immunostained for the proliferation marker Ki-67 and stained for total cell number with DAPI nuclear stain. The ratio of proliferating to total cells (Ki-67/DAPI) was determined. IL-6, but not IL-1, was cytostatic for the C4-2 Par1 parental and C42as1 and C42bs1 subline cells. C4-2 Par1 parental cells showed enhanced cytostasis in response to IL-1 and IL-6 in combination, while the C42as1 and C42bs1 cells did not show enhanced cytostasis in the IL-1/IL-6 combination treatment. Thus, the chronic IL-1 exposure attenuates IL-1/IL-6-induced cytostasis. *n* = 3 biological replicates; error bars = +/−STDEV; *p*-value = * ≤0.05, ** ≤0.005, *** ≤0.0005. Ratios were normalized to vehicle control within each cell line and numerical values indicate percentage change in proliferation.

IL-1 and IL-6 in combination has been shown to synergistically block PCa cell line proliferation [15]. We also set out to investigate if IL-1/IL-6 crosstalk synergistically blocks C4-2 or LNCaP cell proliferation. We treated C4-2 and LNCaP parental cell lines with 25 ng/mL of IL-1α or IL-1β alone, 100 ng/mL of IL-6 alone, or IL-1 and IL-6 in combination for 4 days. Cells were fixed and stained with the proliferation marker Ki-67 and with DAPI to identify viable cells. The ratio of Ki-67-positive proliferating cells to total DAPI-stained cells was determined. The IL-1/IL-6 combination treatment was more cytostatic than either cytokine alone for the C4-2 and LNCaP parental cell lines (Figure 2 and Figure S2), indicating synergistic crosstalk between the IL-1 and IL-6 signaling pathways.

### 3.5. Chronic IL-1 Exposure Attenuates IL-1/IL-6 Intracellular Signaling Crosstalk and Cytostasis

As the combination of IL-1 and IL-6 treatments enhanced intracellular signaling (Figure 1 and Figure S1) and cytostasis (Figure 2 and Figure S2) in the C4-2 and LNCaP PCa parental cell lines, we wanted to determine if chronic IL-1 exposure alters IL-1/IL-6 crosstalk. We treated the C4-2 and LNCaP chronic IL-1 subline cells with 25 ng/mL of IL-1α or IL-1β alone, 100 ng/mL of IL-6 alone, or IL-1 and IL-6 in combination for 4 days. We assessed LCN2, SOD2, STAT3, and phosphorylated STAT3 levels (Figure 1 and Figure S1) and the Ki-67/DAPI ratio (Figure 2 and Figure S2). Compared to parental cells, the chronic IL-1 sublines showed little or no enhanced induction of LCN2, SOD2, STAT3, or phosphorylated STAT3 levels in response to the IL-1/IL-6 combination. Similarly, the chronic IL-1 subline cells showed little or no synergistic cytostatic response to the IL-1/IL-6 combination. Taken together, these data show that chronic IL-1 exposure attenuates IL-1/IL-6 crosstalk, and this is likely due to the loss of IL-1 sensitivity in the chronic IL-1 sublines.

### 3.6. RELA and STAT3 Are Sufficient to Mediate IL-1/IL-6 Cytostatic Crosstalk

**Figure 3:**
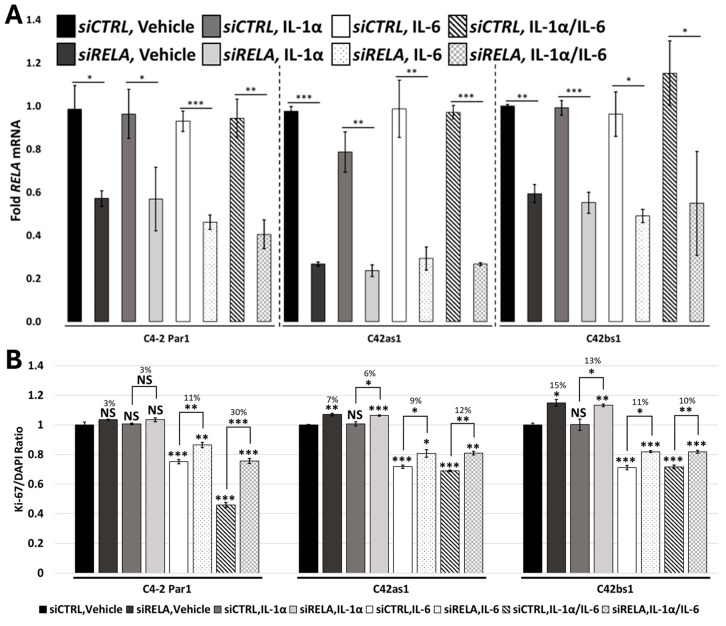
RELA mediates IL-1/IL-6 cytostasis. C4-2 Par1 parental and C42as1 and C42bs1 subline cells were transfected with 70 nM control or *RELA* siRNA 1 day before treatment with vehicle control, 25 ng/mL IL-1α, 100 ng/mL IL-6, or IL-1α/IL-6 in combination for 3 days. (**A**) *RELA* silencing was assessed via RT-qPCR. (**B**) Cells were co-stained for Ki-67 and DAPI to determine the ratio of proliferating cells to total cells. *RELA* silencing attenuated IL-1/IL-6-induced cytostasis in parental cells. *RELA* silencing only marginally attenuated cytostasis in IL-6-treated C4-2 Par1 parental or IL-6- or IL-1/IL-6-treated C42as1 and C42bs1 subline cells. Taken together, RELA mediates IL-1/IL6 cytostasis in C4-2 parental cells, but only marginally in subline cells. *n* = 3 biological replicates; error bars = +/−STDEV; *p*-value = * ≤0.05, ** ≤0.005, *** ≤0.0005. Fold mRNA levels were normalized within the cell line to vehicle control. Ki-67/DAPI ratios were normalized to vehicle control within each subline, and numerical values indicate percentage change in proliferation.

**Figure 4:**
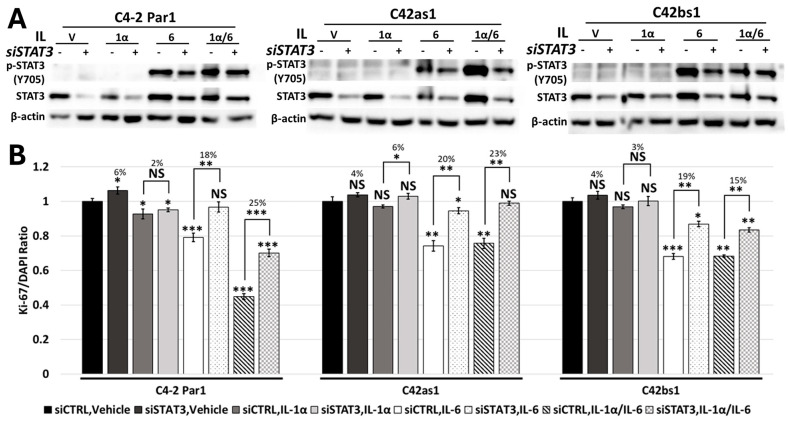
STAT3 mediates IL-1/IL-6 cytostasis. C4-2 Par1 parental and C42as1 and C42bs1 subline cells were transfected with 70 nM control or *STAT3* siRNA 1 day before treatment with vehicle control, 25 ng/mL IL-1α, 100 ng/mL IL-6, or IL-1α/IL-6 in combination for 3 days. (**A**) *STAT3* siRNA silencing was assessed via Western blot. (**B**) Cells were co-stained for Ki-67 and DAPI to determine the ratio of proliferating cells to total cells. *STAT3* silencing attenuated IL-6- and IL-1/IL-6-induced cytostasis in parental and subline cells. Taken together, STAT3 mediates IL-1/IL6 cytostasis in C4-2 parental and subline cells. Taken together, STAT3 mediates IL-1/IL6 cytostasis in C4-2 parental and subline cells. *n* = 3 biological replicates; error bars = +/−STDEV; *p*-value = * ≤0.05, ** ≤0.005, *** ≤0.0005. Ki-67/DAPI ratios are normalized to vehicle control within each subline, and numerical values indicate percent change in proliferation. Original western blots are presented in File S1.

Canonical IL-1 signaling is mediated through RELA/NF-kB transactivation [3], and IL-6 through STAT3 transactivation [14]. Therefore, we siRNA-silenced *RELA* (Figure 3 and Figure S3) or *STAT3* (Figure 4 and Figure S4) in C4-2 and LNCaP parental cell lines treated with 25 ng/mL of IL-1α, 25 ng/mL of IL-1β, or 100 ng/mL of IL-6 alone or in combination. Following treatment, we assessed cell proliferation. *RELA* siRNA silencing was sufficient to rescue IL-1-and/or IL-6-induced cytostasis in C4-2 and LNCaP parental cells, and to a greater extent in the IL-1/IL-6 combination treatment (Figure 3 and Figure S3). In kind, *STAT3* siRNA silencing was sufficient to rescue IL-6-induced cytostasis in parental cells and, to a greater extent, in the IL-1/IL-6 combination treatment (Figure 4 and Figure S4). Furthermore, using LNCaP parental cells, we found that silencing both *RELA* and *STAT3* (Figure S5) rescued IL-1/IL-6-induced cytostasis to a greater extent than silencing *RELA* (Figure S3) or *STAT3* (Figure S4) alone. Taken together, *RELA* and *STAT3* mediate IL-1/IL-6 cytostatic crosstalk.

### 3.7. Chronic IL-1 Exposure Attenuates RELA-Dependent IL-1/IL-6 Cytostatic Crosstalk

As previously stated, IL-6 and IL-1/IL-6 induce cytostasis in the C4-2 and LNCaP chronic IL-1 sublines (Figure 2 and Figure S2). However, the sublines show little or no cytostasis in response to IL-1 (Figure 2 and Figure S2). As such, *RELA* siRNA silencing led to little or no rescue of cytostasis in the sublines treated with IL-1, IL-6, or IL-1/IL-6 in combination (Figure 3 and Figure S3). On the other hand, *STAT3* siRNA silencing caused comparable rescue of cytostasis in IL-6- and IL-1/IL-6-treated subline cells (Figure 4 and Figure S4). These data suggest that IL-6/ STAT3 signaling, but not IL-1/RELA signaling, remains functional in the chronic IL-1 sublines. As a consequence, crosstalk between IL-1/RELA and IL-6/STAT3 is attenuated in the sublines.

## 4. Discussion

We previously reported that PCa cells acquire stable changes in gene expression following chronic IL-1 exposure that may contribute to disease progression [12,22]. For example, gene ontology predicts that chronic IL-1 exposure alters cytokine response [22]. Given that there are multiple pro-tumorigenic inflammatory cytokines signaling in the PCa tumor microenvironment [10,11], including IL-6 [14], we wanted to determine if chronic IL-1 exposure alters cell response to other cytokines. For example, we found that chronic IL-1 exposure reduces sensitivity to acute IL-1 or TNFα treatment [12], both which signal through NF-kB [3]. In this report, we assessed the effect of chronic IL-1 exposure on PCa cell response to IL-6, which signals through STAT3 [14].

As previously observed for LNCaP and MDA-PCa-2b PCa cell lines [12,22] chronic IL-1 exposure caused C4-2 cells to evolve insensitivity to acute IL-1 exposure (Figure 1 and Figure S1). While IL-1R1 receptor levels are comparable in the parental and subline cells ([12], Figure S1C), it is possible that IL-1 signaling is dampened in the sublines due to reduced receptor/ligand affinity and/or an aberrant regulation of the downstream NF-kB signaling cascade. While the mechanism(s) of chronic IL-1-induced IL-1 insensitivity requires further investigation, our data presented in this and other [12] studies clearly show that chronic IL-1 exposure alters how cells respond to other cytokines. In this study we show that chronic IL-1 exposure dampens IL-1/RELA and IL-6/STAT3 synergy.

Others have shown that IL-1 and IL-6 in combination synergistically block PCa cell line proliferation [15]. However, we find that cells chronically exposed to IL-1 show attenuated IL-1/IL-6 cytostatic response (Figure 2 and Figure S2). This attenuation is likely due to their acquired IL-1 insensitivity (Figure 1 and Figure S1). As such, *RELA* siRNA was not sufficient to rescue cytostasis in IL-1/IL-6-treated sublines (Figure 3 and Figure S3). Thus, chronic IL-1 exposure may select for cells that have a proliferative advantage in the cytokine-rich tumor microenvironment.

In vivo studies have demonstrated that blocking the IL-1 receptor bypasses immunosuppression to reduce tumor growth [23,24]. FDA-approved anti-arthritic drugs that inhibit IL-1 activity, such as canakinumab and anakinra, are under investigation in clinical trials to combat cancer progression [3,25,26,27,28]. However, if cell populations have become desensitized to IL-1 signaling via chronic IL-1 exposure, these treatment methods may not be as efficacious as previously hoped.

IL-6 has been linked to PCa progression [14]. As such, IL-6 receptor inhibitors, such as tocilizumab, have been proposed as possible therapeutics for cancer treatment [29,30,31]. Both parental and subline cells increased STAT3 phosphorylation (Figure 1 and Figure S1) and showed cytostasis in response to IL-6 or IL-1/IL-6 (Figure 2 and Figure S2). While *RELA* siRNA was not sufficient to rescue cytostasis in IL-1/IL-6-treated sublines (Figure 3 and Figure S3), *STAT3* siRNA was sufficient (Figure 4 and Figure S4). Thus, IL-6-targeted therapies that inhibit STAT3 activity could still be effective against cancer cells chronically exposed to IL-1.

Both IL-1 [16,17] and IL-6 [19,32] have been reported to induce neuronal-like transdifferentiation in PCa cell lines. The neuronal-like morphology is accompanied by cytostasis, which would render the cells insensitive to cell cycle-targeting chemoradiation [19]. But silencing IL-1/RELA (Figure 3 and Figure S3) and/or IL-6/STAT3 (Figure 4 and Figure S4) activity can reverse cytostasis in the parental cells and/or subline cells, thereby theoretically rendering the cells sensitive to chemoradiation.

**Figure 5:**
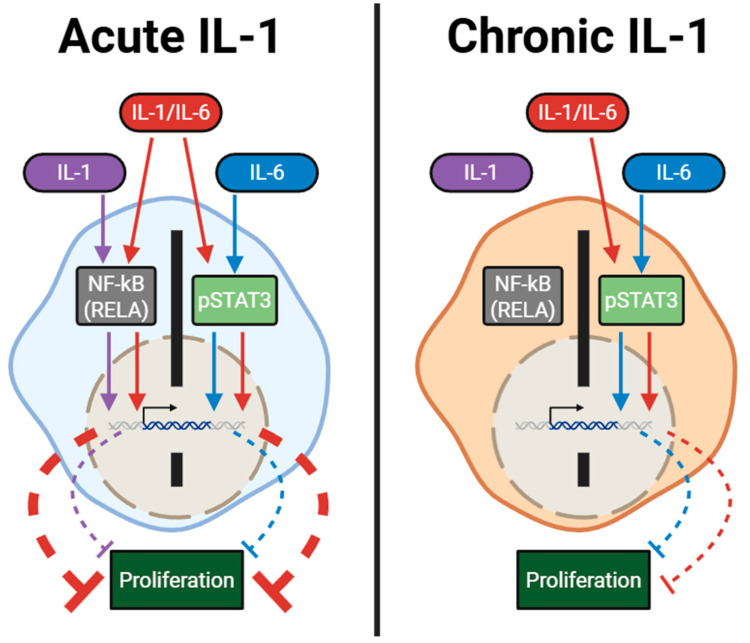
Model. Acute IL-1 and IL-6 signal through *RELA* and *STAT3*, respectively, to inhibit proliferation in PCa cell lines. IL-1/IL-6 in combination has an enhanced cytostatic effect on cells and signal through both *RELA* and *STAT3* transcription factors to regulate proliferation. Chronic IL-1 selects for cells that have lost sensitivity to IL-1 but not IL-6 signaling, resulting in cells that respond comparably to IL-6 treatment alone or in combination with IL-1. Image created in BioRender. Yamauchi, S.A. (2025) https://BioRender.com/29s4q3w.

We show that chronic exposure to IL-1 alters how cells respond to other inflammatory cytokines such as TNFα [12] and IL-6 (this report, Figure 5). Our data suggest that chronic IL-1 exposure selects for cells that are insensitive to IL-1 or to RELA inhibitors but still maintain sensitivity to IL-6 or to STAT3 inhibitors. And while chronic IL-1 exposure selects for cells that dampen IL-1/IL-6 synergy, these cells would presumably be more susceptible to chemoradiation cytotoxicity. Thus, deciding on the best therapeutic approach requires understanding the integration of inflammatory and other signaling molecules in the tumor microenvironment.

## 5. Conclusions

In conclusion, we have found that (1) chronic IL-1 exposure causes PCa cell lines to lose or attenuate sensitivity to acute IL-1 signaling; (2) IL-1 and IL-6 synergistically inhibit proliferation in C4-2 and LNCaP PCa cells, but this synergy is lost or attenuated in the chronic IL-1 sublines; and (3) IL-1/IL-6 synergistic cytostasis is mediated via *RELA* and *STAT3*. Thus, a cell population existing within the tumor microenvironment with lost or attenuated response to IL-1 signaling could impact the efficacy of IL-1-targeted therapies or therapies that target crosstalk signaling with other cytokines and signaling molecules.

## Data Availability

The original contributions presented in this study are included in the article. Further inquiries can be directed to the corresponding author.

## Supplementary Materials

The following supporting information can be downloaded at https://www.mdpi.com/article/10.3390/cancers17233778/s1, Figure S1: Chronic IL-1 exposure attenuates IL-1 and IL-1/IL-6 intracellular signaling.; Figure S2: Chronic IL-1 exposure attenuates IL-1/IL-6-induced cytostasis.; Figure S3: RELA mediates IL-1/IL-6 cytostasis.; Figure S4: STAT3 mediates IL-1/IL-6 cytostasis.; Figure S5: RELA and STAT3 mediate IL-1/IL-6 cytostasis. File S1: Original western blots.

## Abbreviations

The following abbreviations are used in this manuscript:

PCa	Prostate Cancer
IL-1α	Interleukin-1 alpha
IL-1β	Interleukin-1 beta
IL-6	Interleukin-6
C4-2 Par1	C4-2 Parental subline 1
C4-2 Par2	C4-2 Parental subline 2
C42as1	C4-2 chronic IL-1α subline 1
C42as2	C4-2 chronic IL-1α subline 2
C42bs1	C4-2 chronic IL-1β subline 1
C42bs2	C4-2 chronic IL-1β subline 2
LNCaP-1	LNCaP Parental subline 1
LNas1	LNCaP chronic IL-1α subline 1
LNbs1	LNCaP chronic IL-1β subline 1
