# Supplementary material for: Chronic IL-1 Exposure Attenuates RELA- and STAT3-Dependent Synergistic Cytokine Signaling in Prostate Cancer Cell Lines
Source: Cancers (Basel). 2025 Nov 26;17(23):3778. doi: 10.3390/cancers17233778 (PMC12691196; doi:10.3390/cancers17233778)
Supplement: Supplementary file 1 [file cancers-17-03778-s001.zip › cancers-3991036-File S1.pdf]

FIGURE 1B

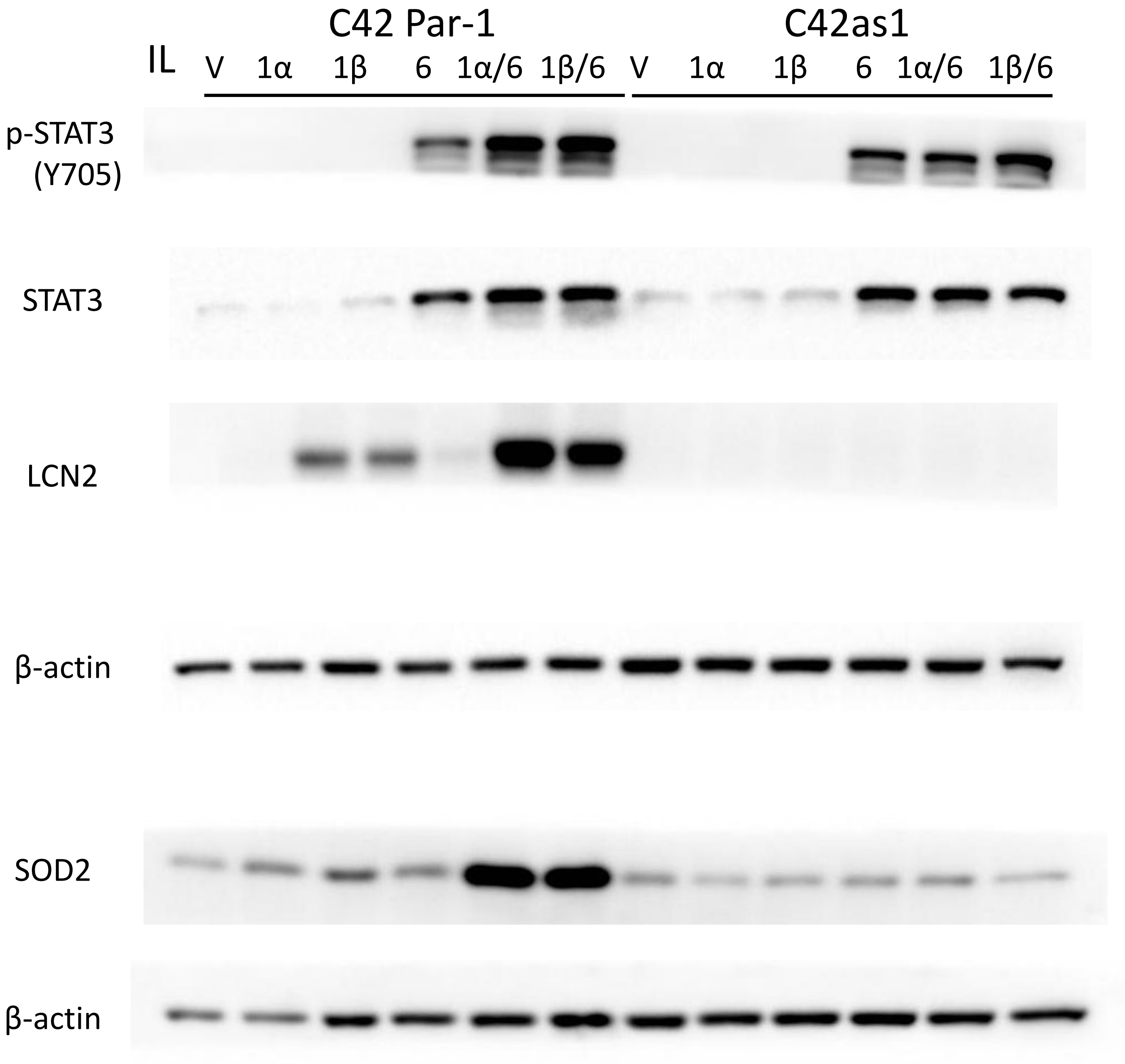

C42 Par-1
C42as1
IL
V 1α 1β 6 1α/6 1β/6 V 1α 1β 6 1α/6 1β/6
p-STAT3 (Y705)
STAT3
LCN2
β-actin
SOD2
β-actin

FIGURE 1B

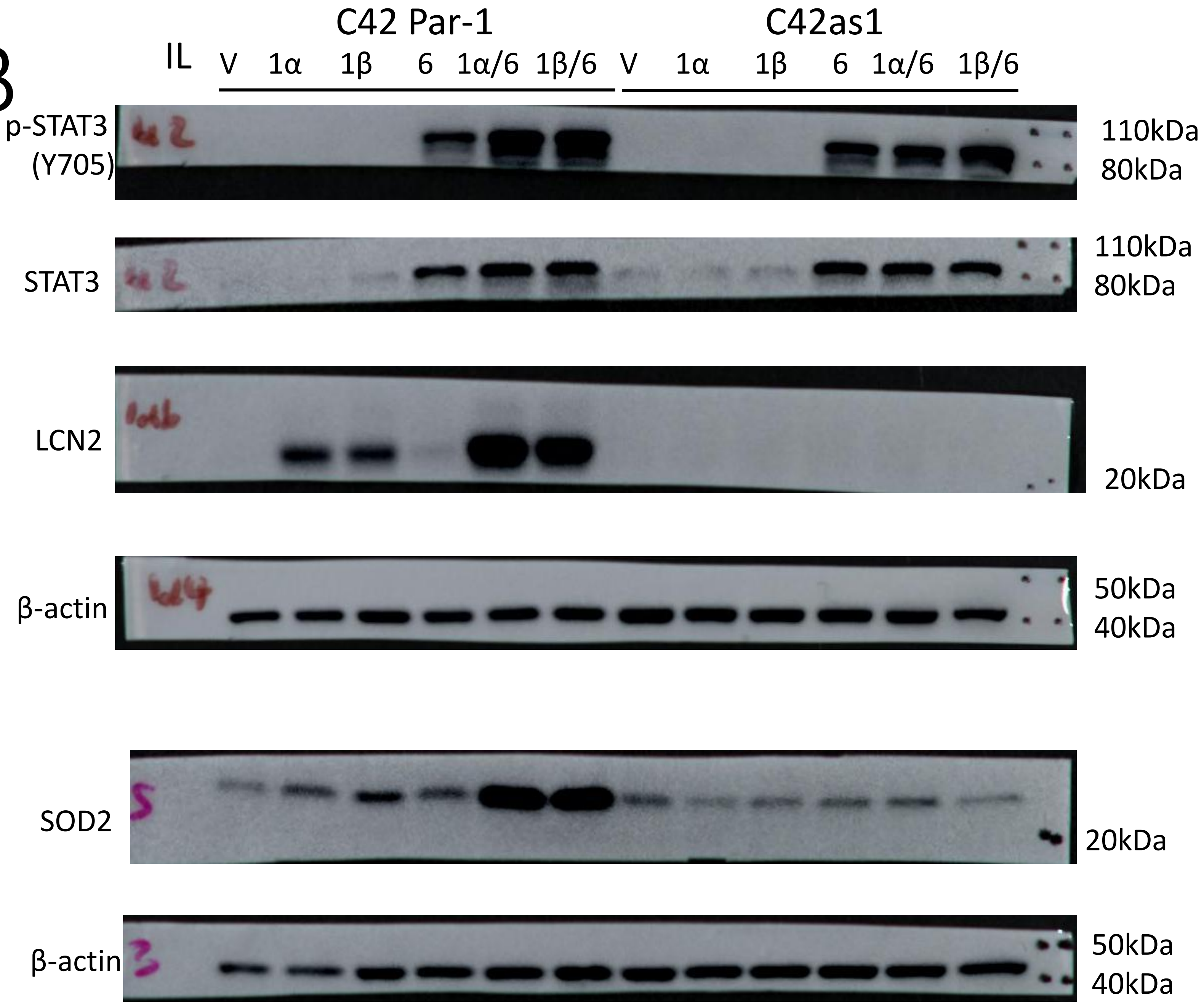

C42 Par-1
C42as1
IL
V 1α 1β 6 1α/6 1β/6
V 1α 1β 6 1α/6 1β/6
p-STAT3 (Y705)
110kDa
80kDa
STAT3
110kDa
80kDa
LCN2
20kDa
β-actin
50kDa
40kDa
SOD2
20kDa
β-actin
50kDa
40kDa

FIGURE 1B

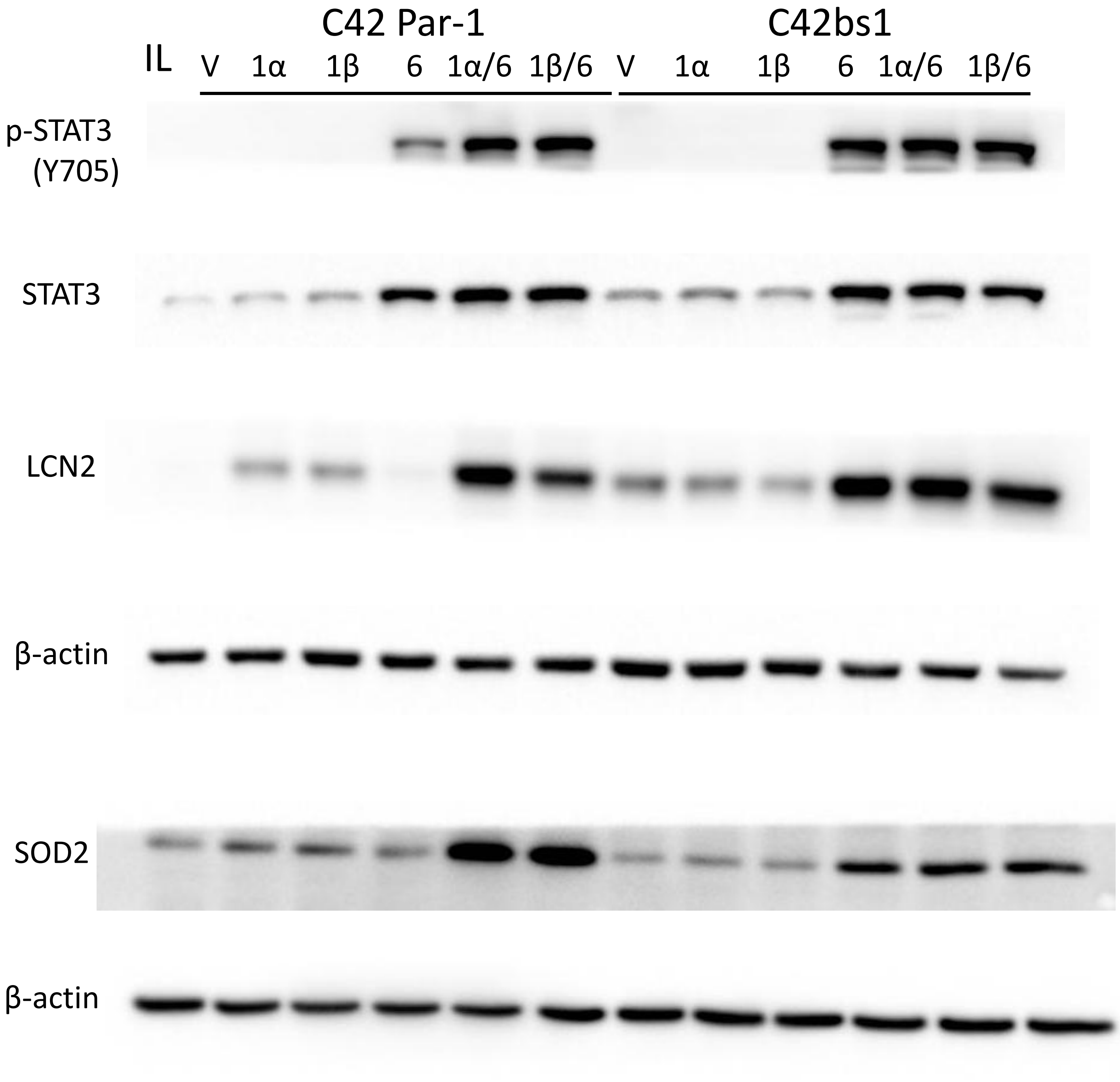
C42 Par-1
C42bs1
IL
V
1α
1β
6
1α/6
1β/6
V
1α
1β
6
1α/6
1β/6
p-STAT3 (Y705)
STAT3
LCN2
β-actin
SOD2
β-actin

FIGURE 1B

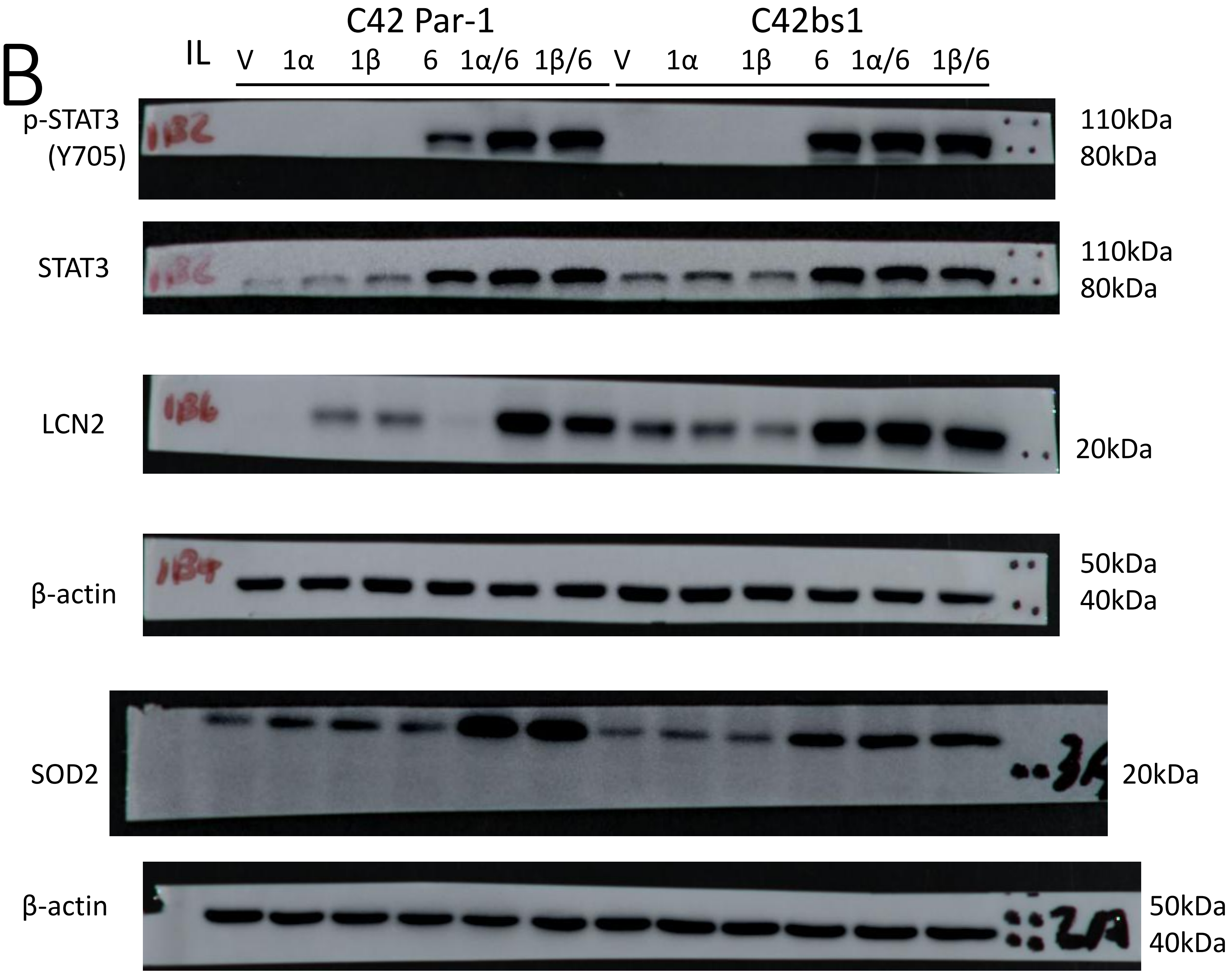
C42 Par-1
C42bs1
IL
V 1α 1β 6 1α/6 1β/6
V 1α 1β 6 1α/6 1β/6
p-STAT3 (Y705)
110kDa
80kDa
STAT3
110kDa
80kDa
LCN2
20kDa
β-actin
50kDa
40kDa
SOD2
20kDa
β-actin
50kDa
40kDa

FIGURE S1C

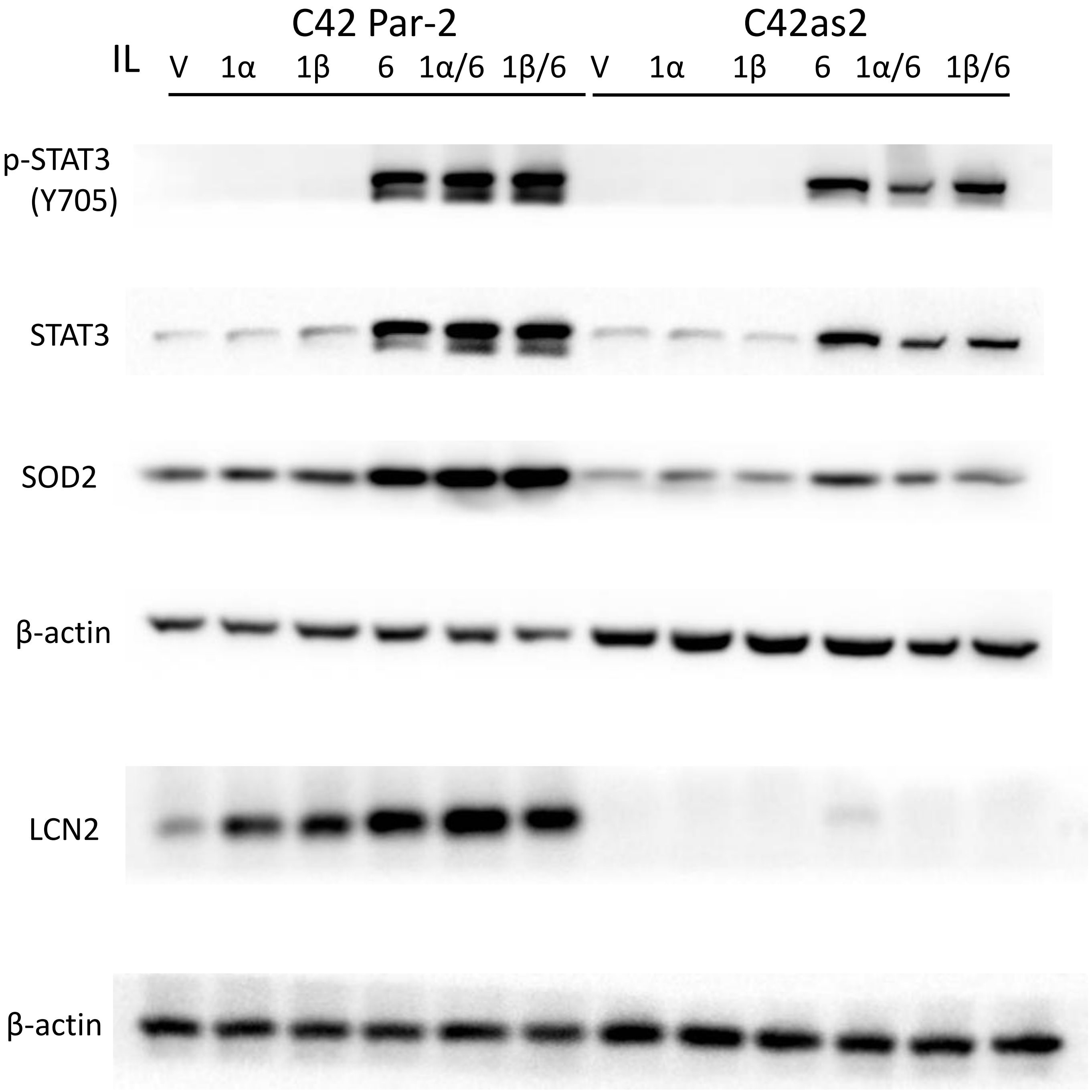
C42 Par-2
C42as2
IL
V 1α 1β 6 1α/6 1β/6
V 1α 1β 6 1α/6 1β/6
p-STAT3 (Y705)
STAT3
SOD2
β-actin
LCN2
β-actin

FIGURE S1C

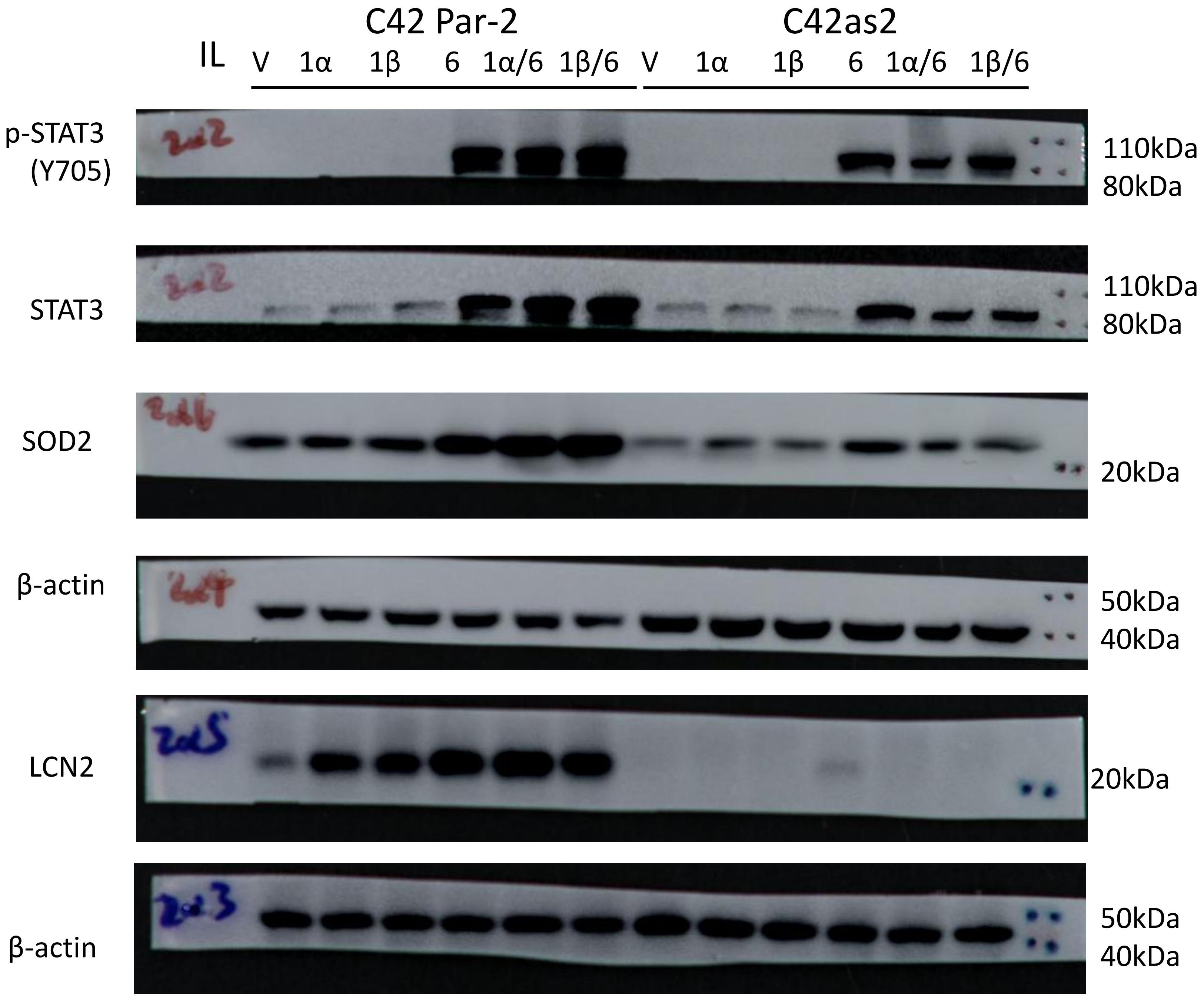
IL
C42 Par-2
C42as2
V 1α 1β 6 1α/6 1β/6 V 1α 1β 6 1α/6 1β/6
p-STAT3
(Y705)
STAT3
SOD2
β-actin
LCN2
β-actin
110kDa
80kDa
110kDa
80kDa
20kDa
50kDa
40kDa
20kDa
50kDa
40kDa

FIGURE S1C

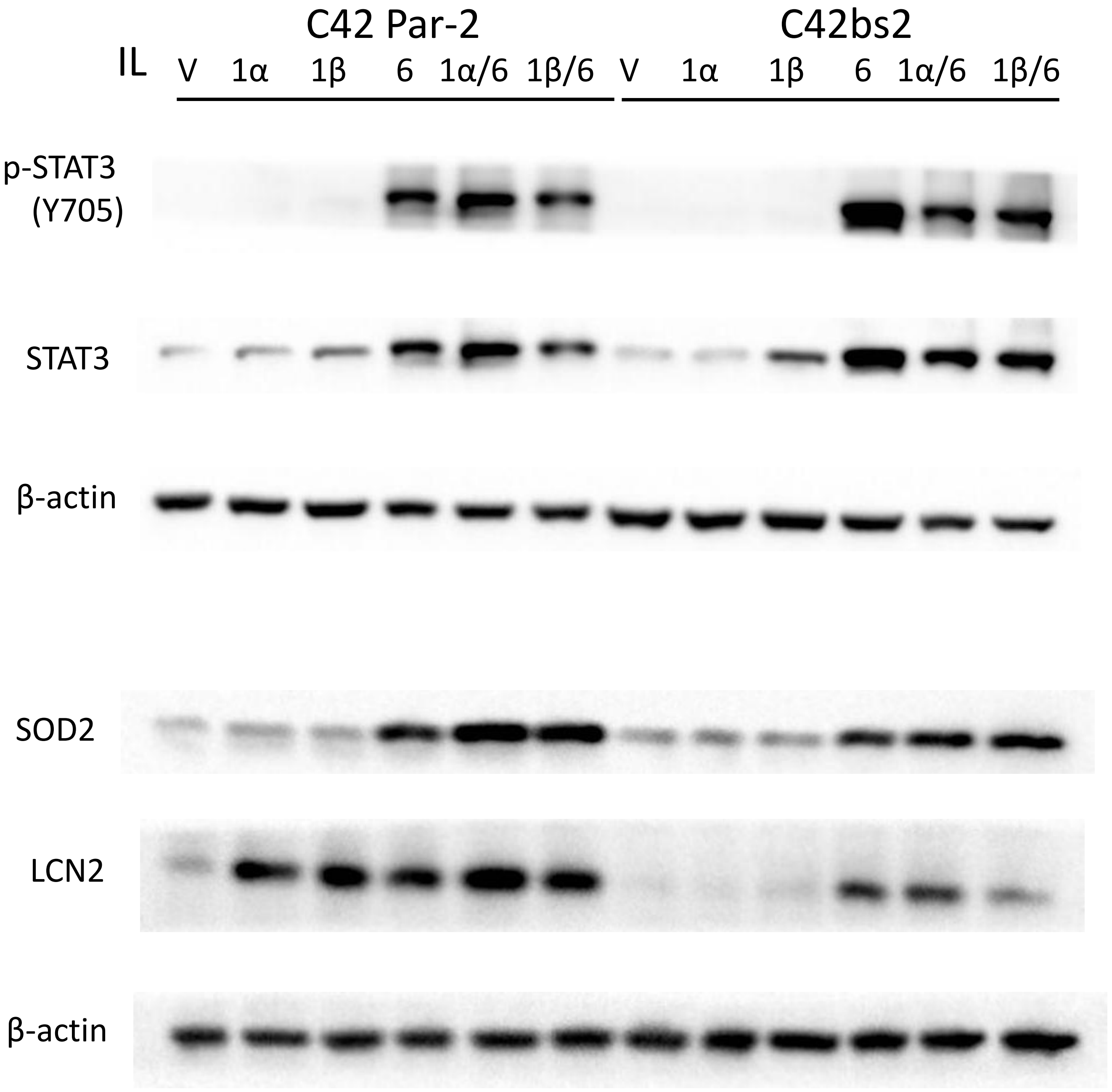

C42 Par-2
C42bs2
IL
V 1α 1β 6 1α/6 1β/6 V 1α 1β 6 1α/6 1β/6
p-STAT3 (Y705)
STAT3
β-actin
SOD2
LCN2
β-actin

FIGURE S1C

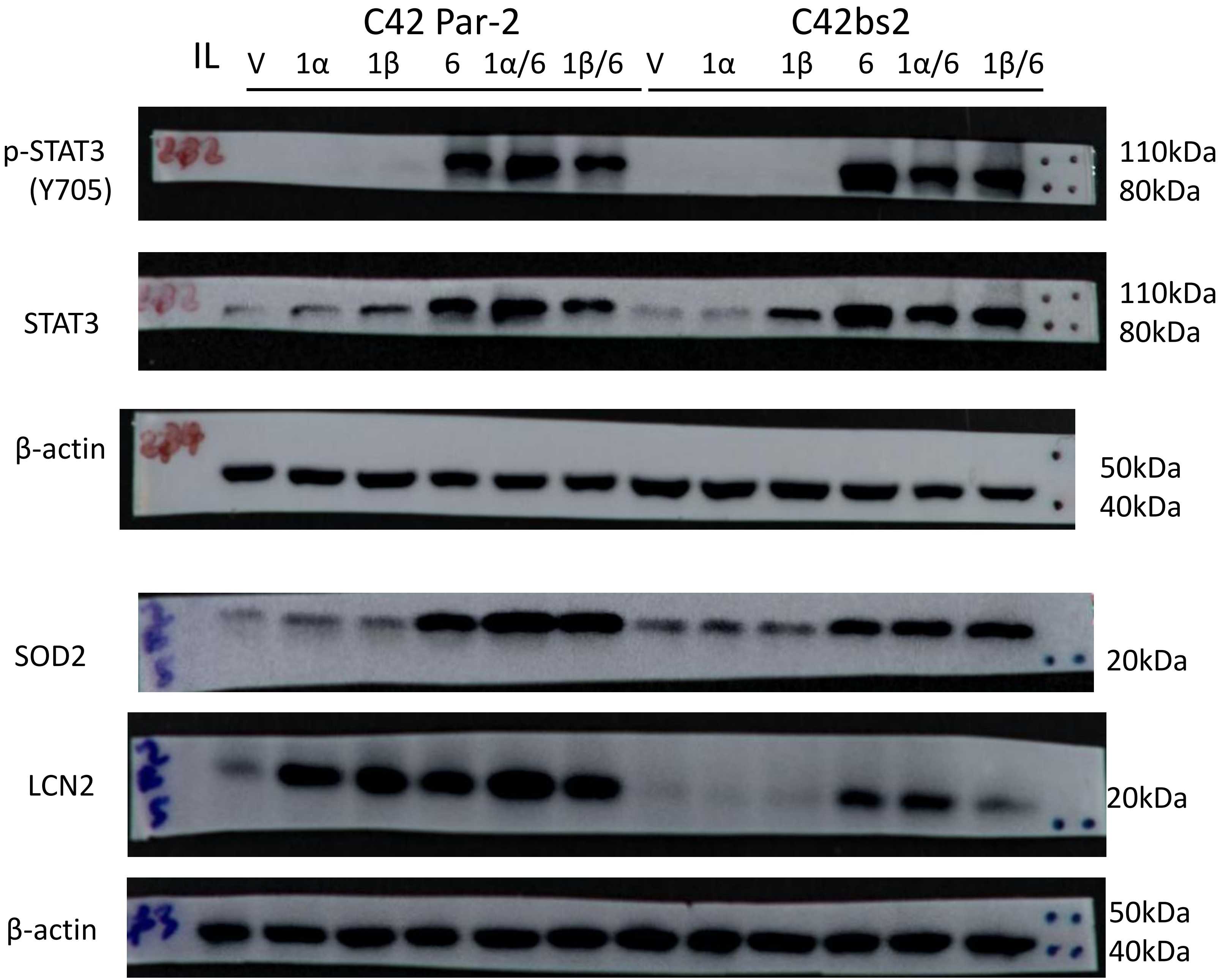
C42 Par-2
C42bs2
IL
V 1α 1β 6 1α/6 1β/6 V 1α 1β 6 1α/6 1β/6
p-STAT3 (Y705)
110kDa
80kDa
STAT3
110kDa
80kDa
β-actin
50kDa
40kDa
SOD2
20kDa
LCN2
20kDa
β-actin
50kDa
40kDa

FIGURE S1D

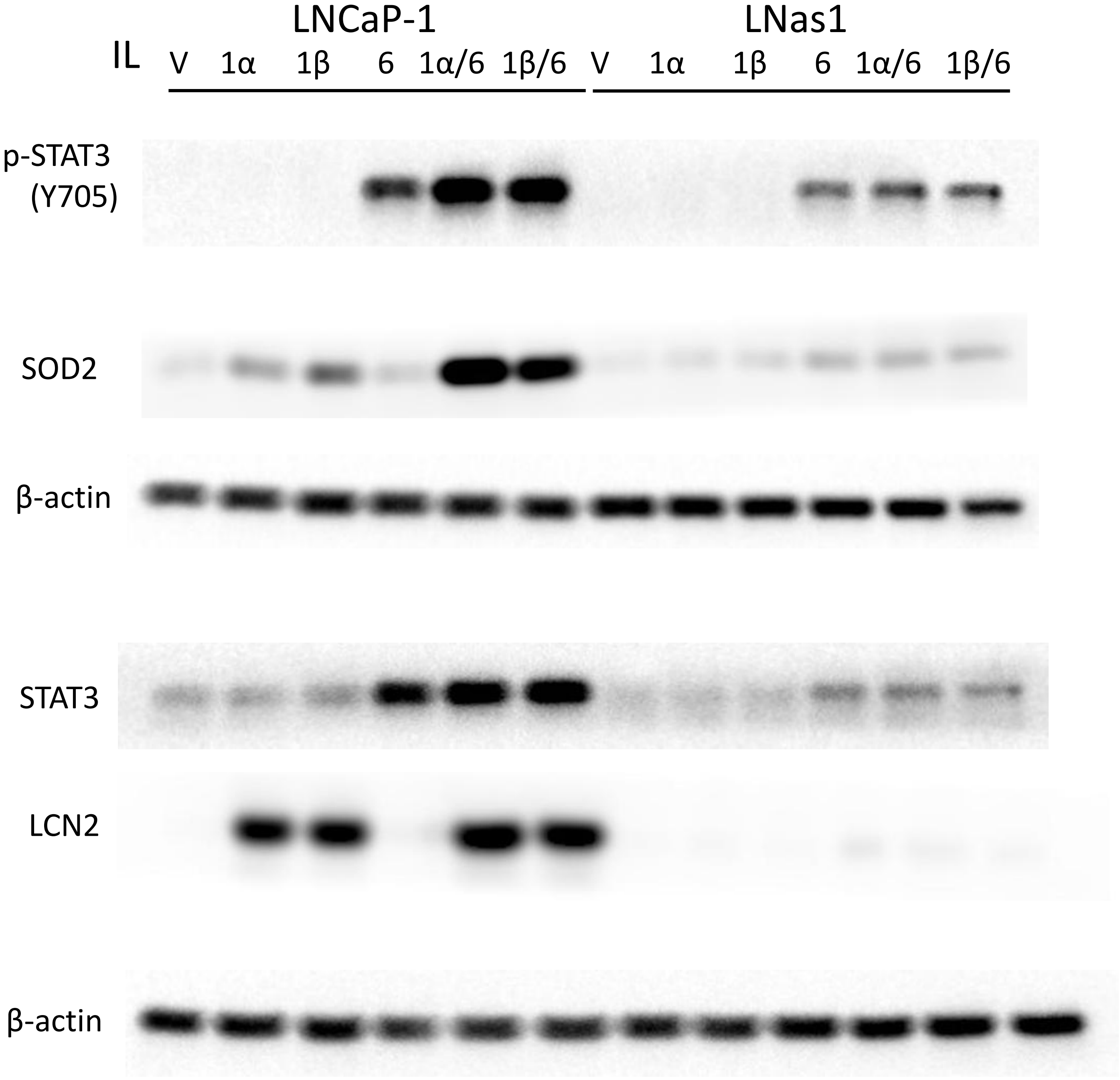
LNCaP-1
LNas1
IL
V 1α 1β 6 1α/6 1β/6
V 1α 1β 6 1α/6 1β/6
p-STAT3 (Y705)
SOD2
β-actin
STAT3
LCN2
β-actin

FIGURE S1D

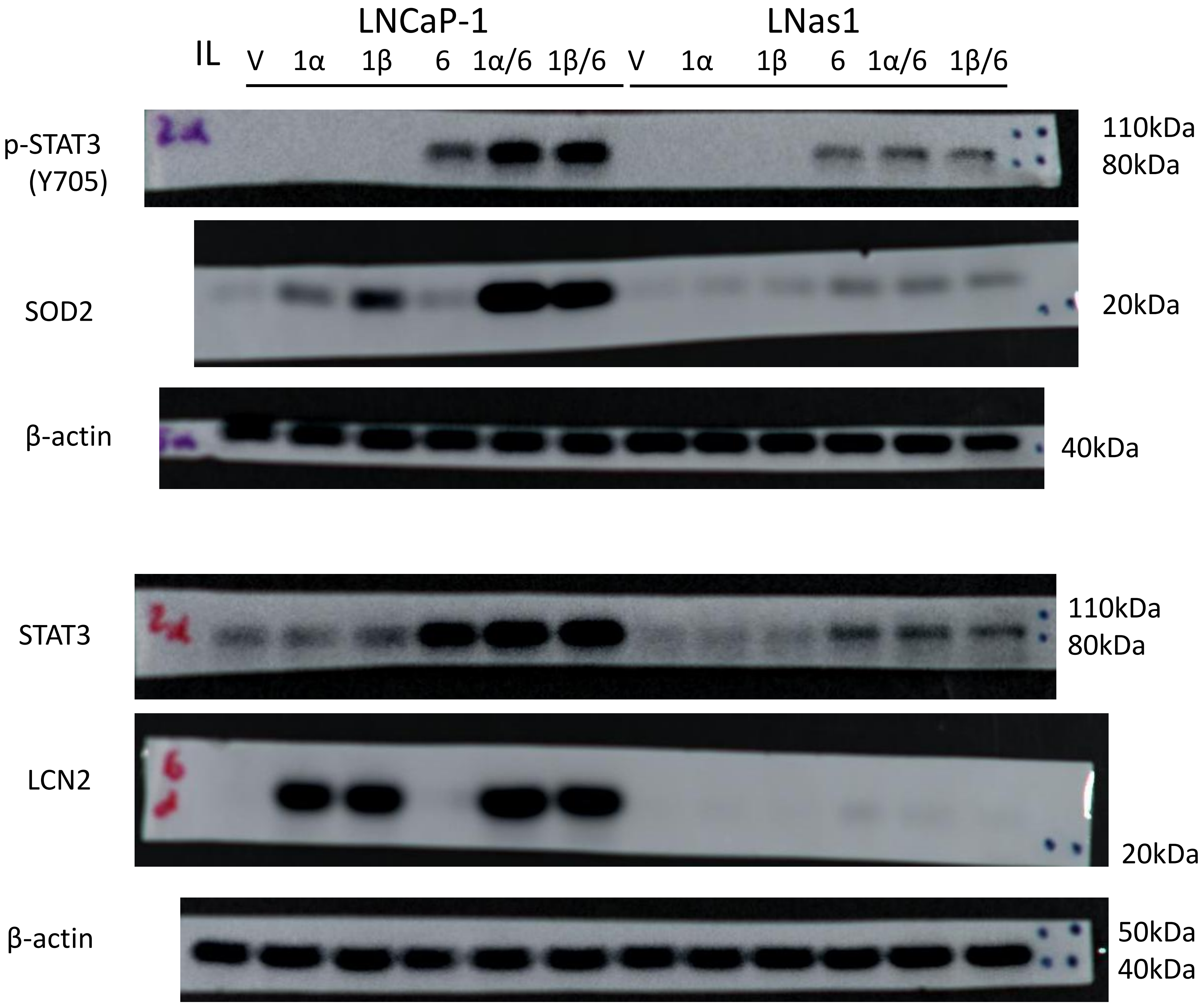
LNCaP-1
LNas1
IL
V 1α 1β 6 1α/6 1β/6
V 1α 1β 6 1α/6 1β/6
p-STAT3 (Y705)
110kDa
80kDa
SOD2
20kDa
β-actin
40kDa
STAT3
110kDa
80kDa
LCN2
20kDa
β-actin
50kDa
40kDa

FIGURE S1D

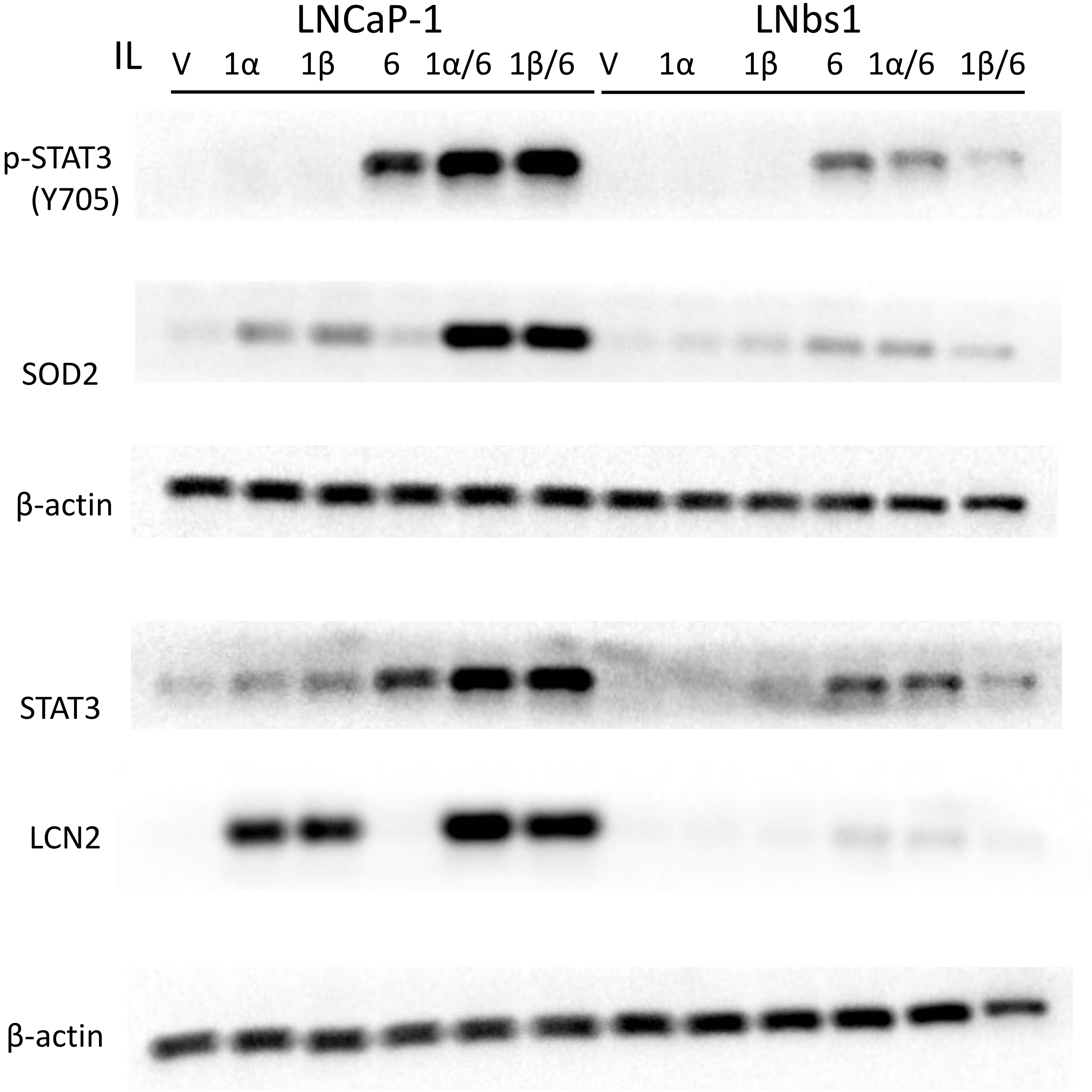

LNCaP-1
LNbs1
IL
V
1α
1β
6
1α/6
1β/6
V
1α
1β
6
1α/6
1β/6
p-STAT3 (Y705)
SOD2
β-actin
STAT3
LCN2
β-actin

FIGURE S1D

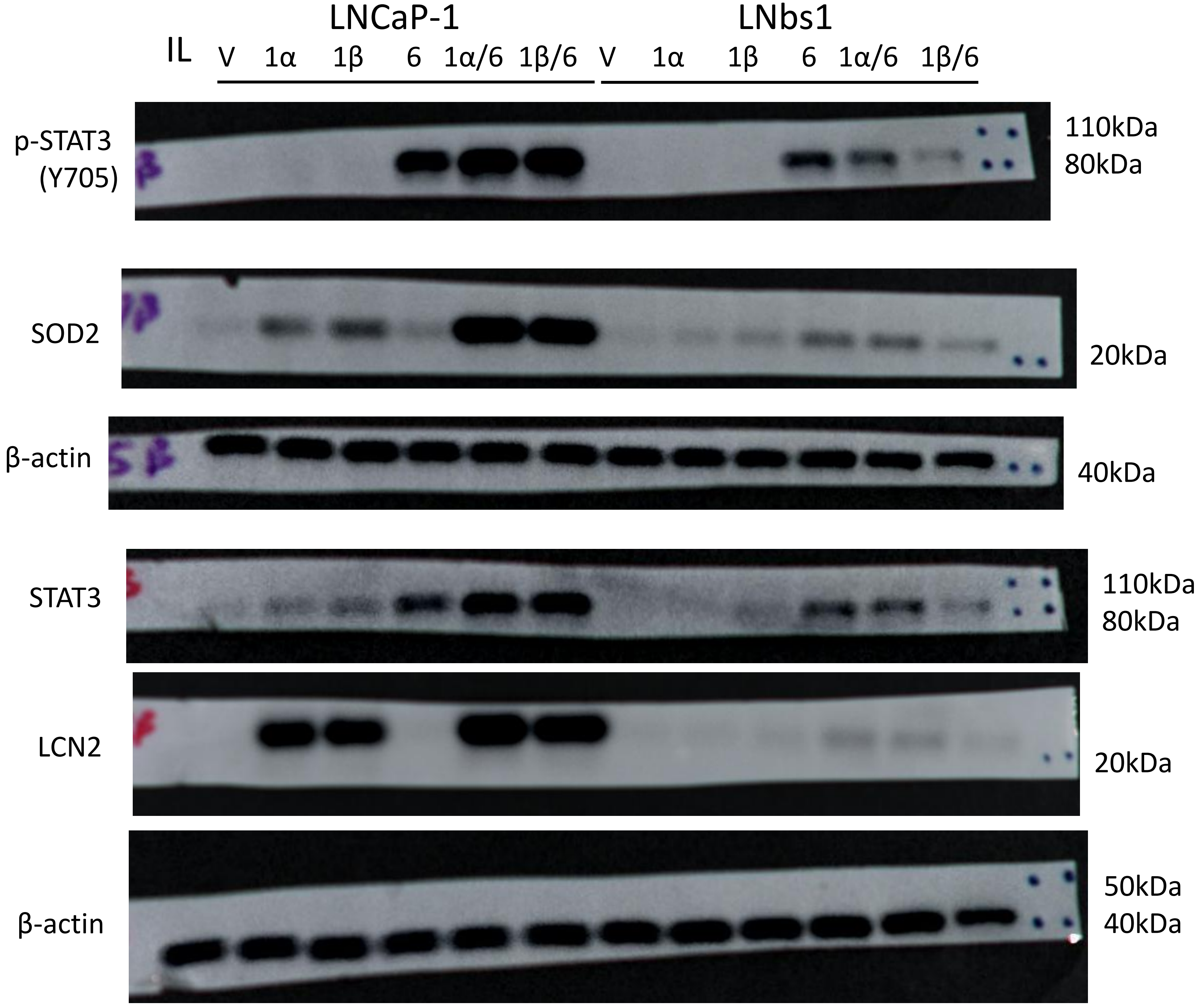
LNCaP-1
LNbs1
IL
V 1α 1β 6 1α/6 1β/6
V 1α 1β 6 1α/6 1β/6
p-STAT3 (Y705)
SOD2
β-actin
STAT3
LCN2
β-actin
110kDa
80kDa
20kDa
40kDa
110kDa
80kDa
20kDa
50kDa
40kDa

FIGURE 4A

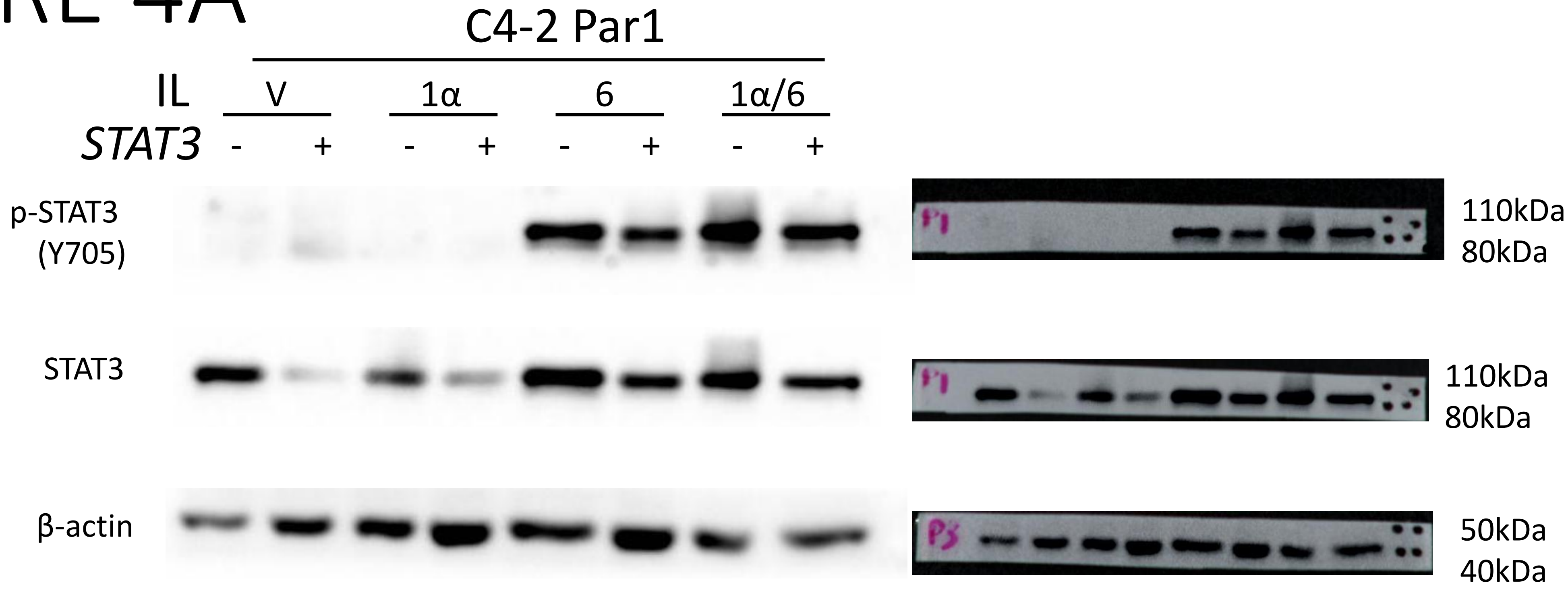
C4-2 Par1
IL
V
1α
6
1α/6
STAT3
-
+
-
+
-
+
-
+
p-STAT3
(Y705)
STAT3
β-actin
110kDa
80kDa
110kDa
80kDa
50kDa
40kDa

FIGURE 4A

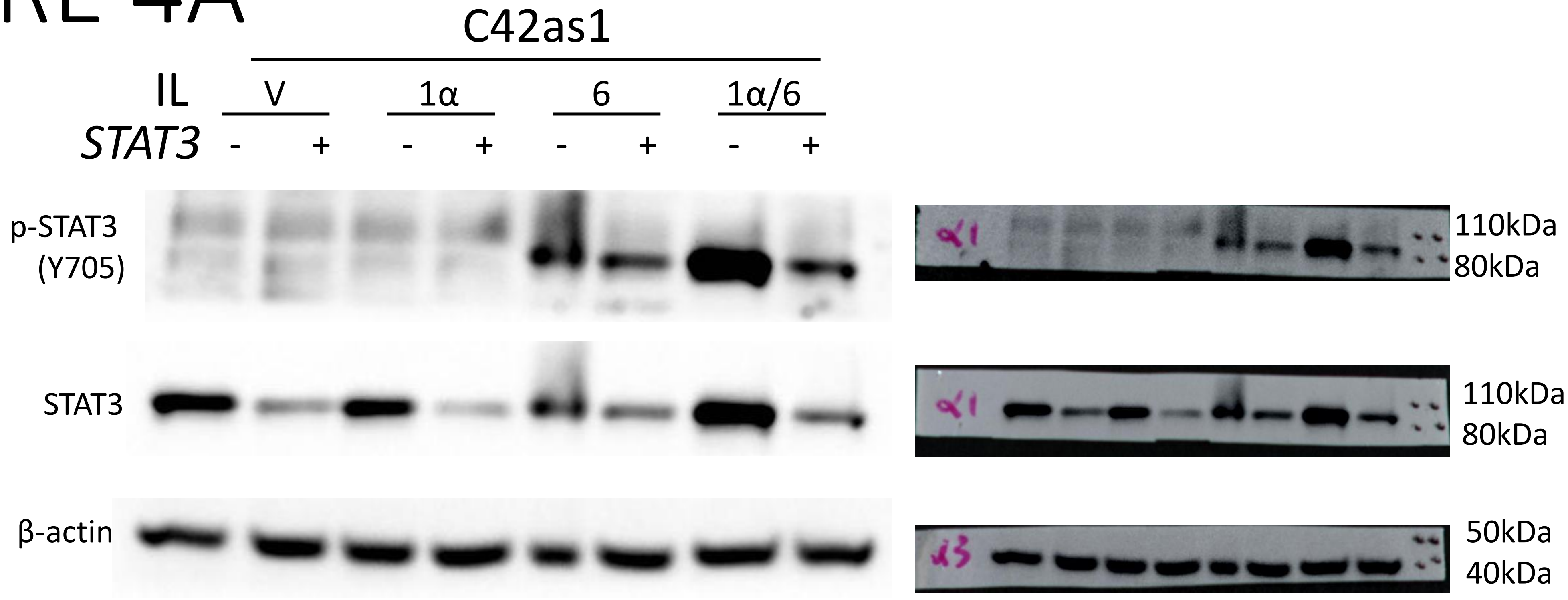
C42as1
IL
V
1α
6
1α/6
STAT3
- + - + - + - +
p-STAT3
(Y705)
STAT3
β-actin
110kDa
80kDa
110kDa
80kDa
50kDa
40kDa

FIGURE 4A

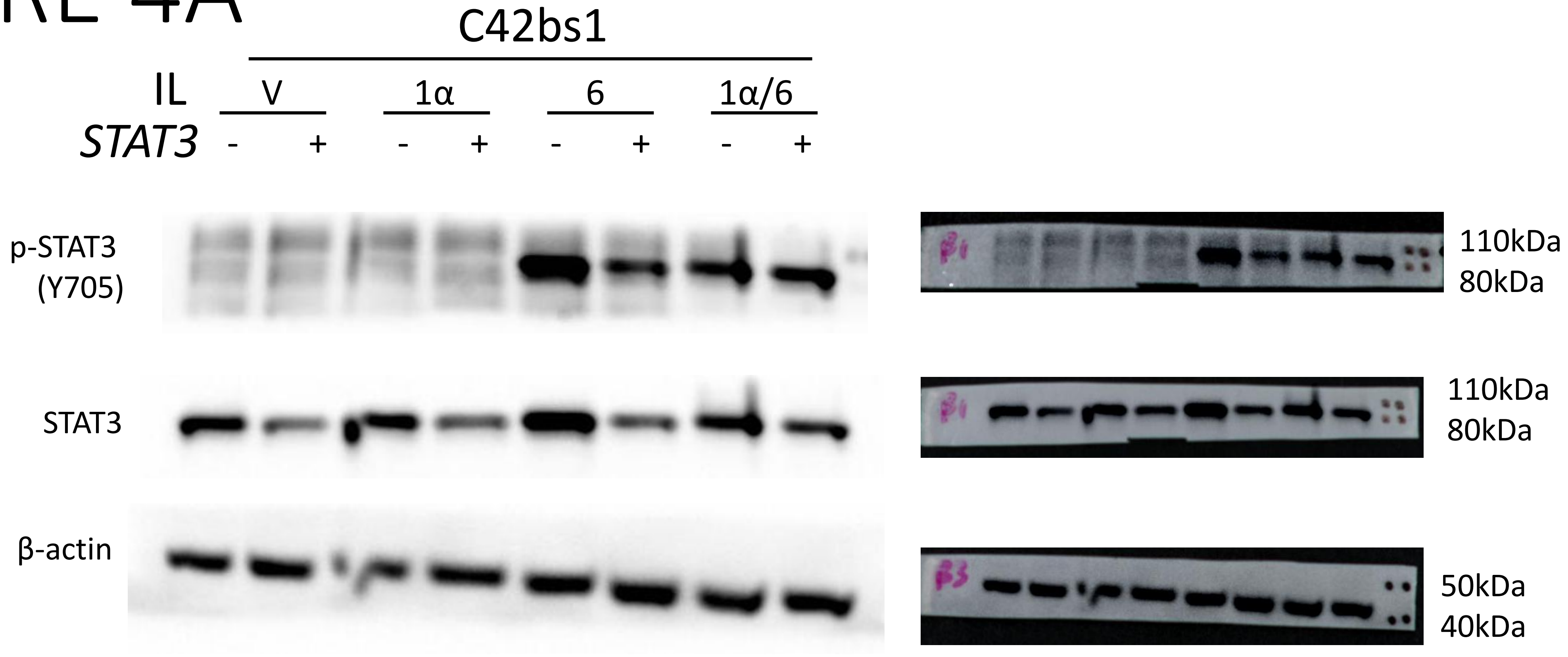
C42bs1
IL
V
1α
6
1α/6
STAT3
- + - + - + - +
p-STAT3
(Y705)
STAT3
β-actin
110kDa
80kDa
110kDa
80kDa
50kDa
40kDa

FIGURE S4A

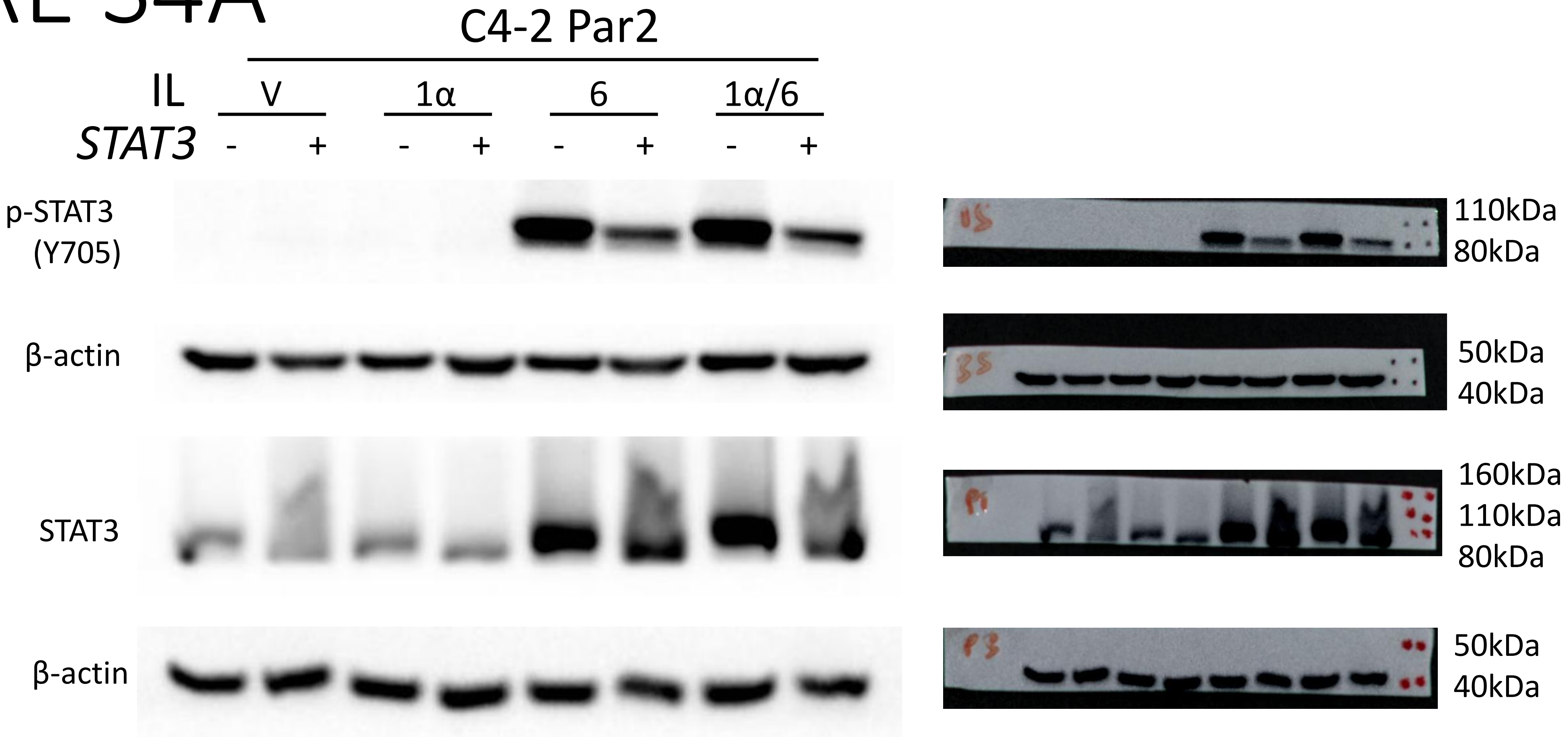
C4-2 Par2
IL
V
1α
6
1α/6
STAT3
- + - + - + - +
p-STAT3
(Y705)
β-actin
STAT3
β-actin
110kDa
80kDa
50kDa
40kDa
160kDa
110kDa
80kDa
50kDa
40kDa

FIGURE S4A

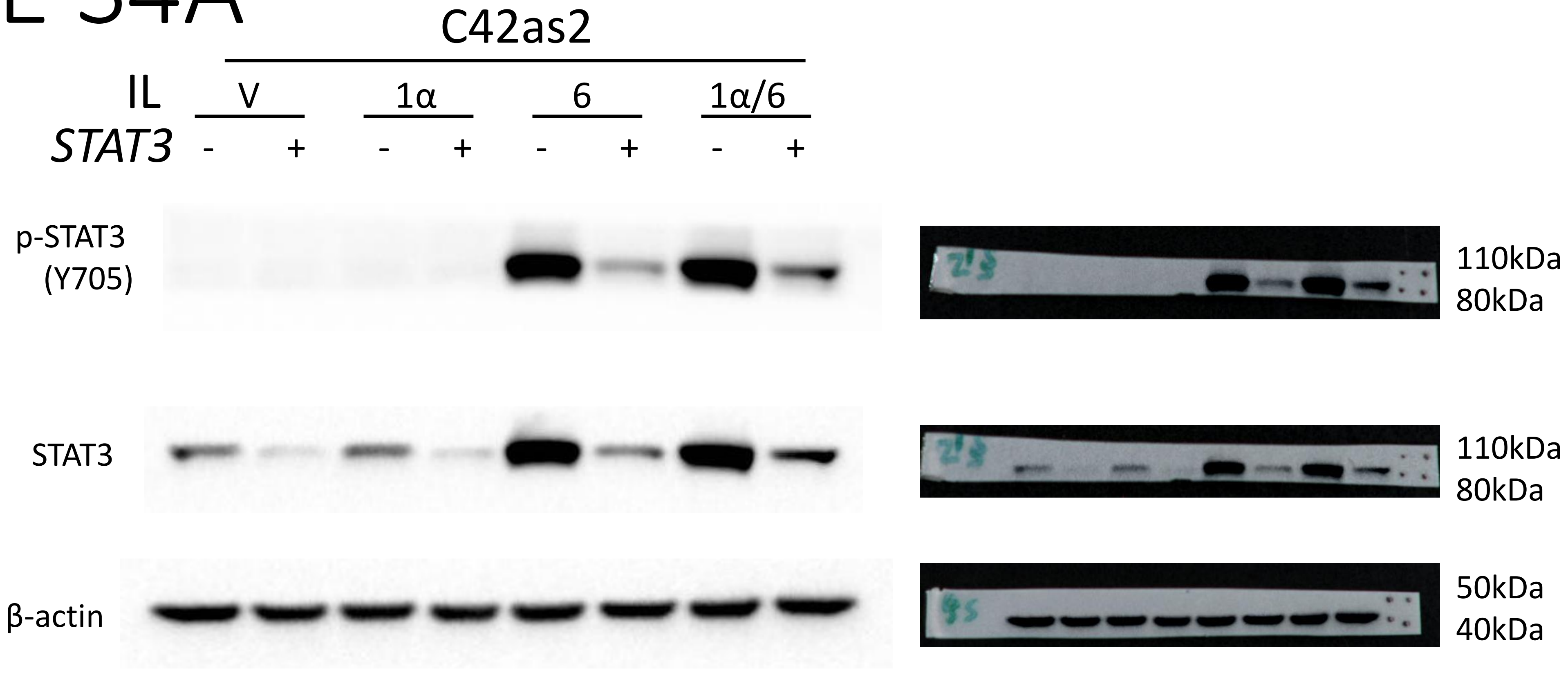
C42as2
IL
V
1α
6
1α/6
STAT3
- + - + - + - +
p-STAT3
(Y705)
STAT3
β-actin
110kDa
80kDa
110kDa
80kDa
50kDa
40kDa

FIGURE S4A

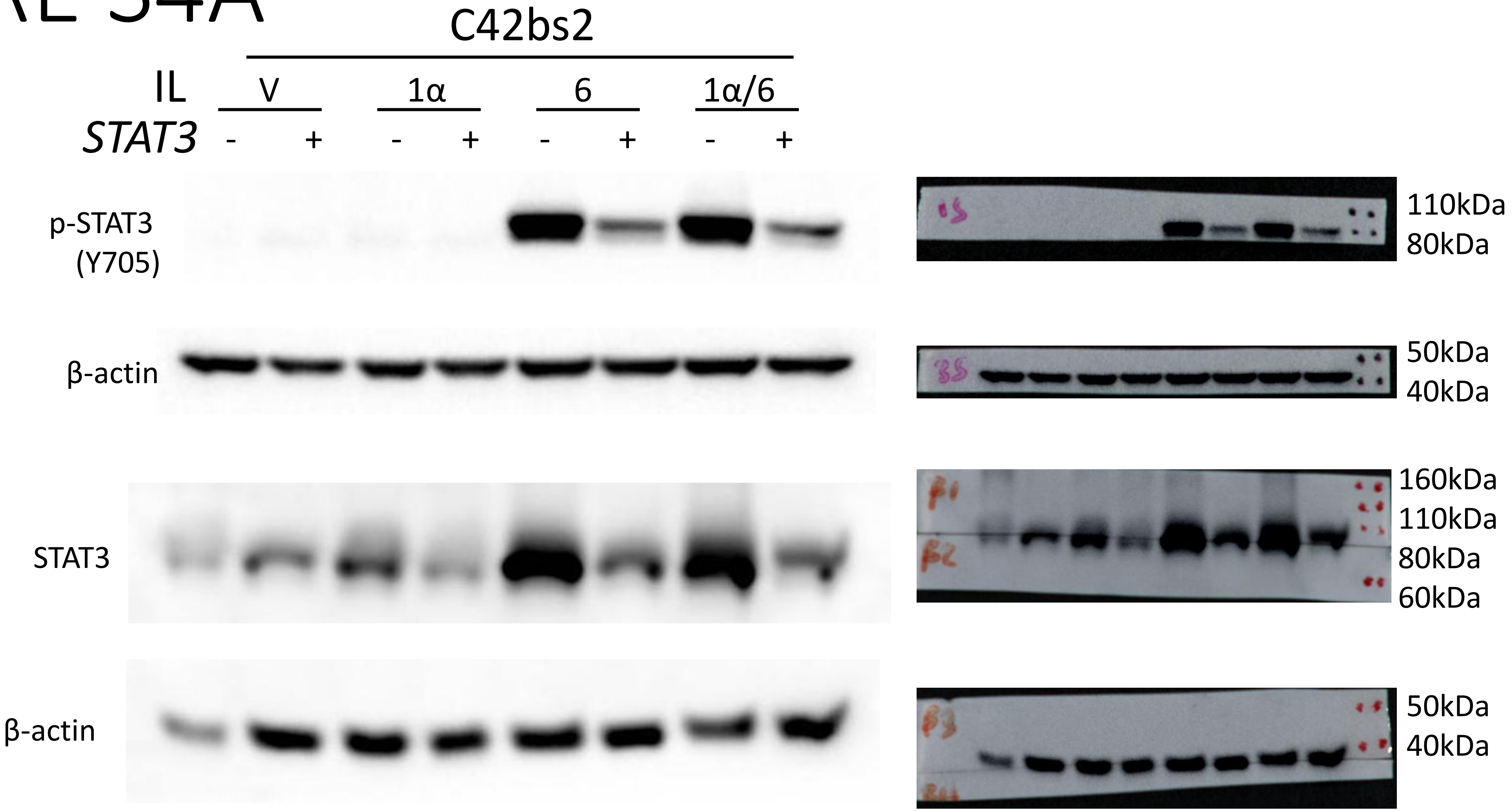
C42bs2
IL
V
1α
6
1α/6
STAT3
- + - + - + - +
p-STAT3 (Y705)
β-actin
STAT3
β-actin
110kDa
80kDa
50kDa
40kDa
160kDa
110kDa
80kDa
60kDa
50kDa
40kDa

FIGURE S4A

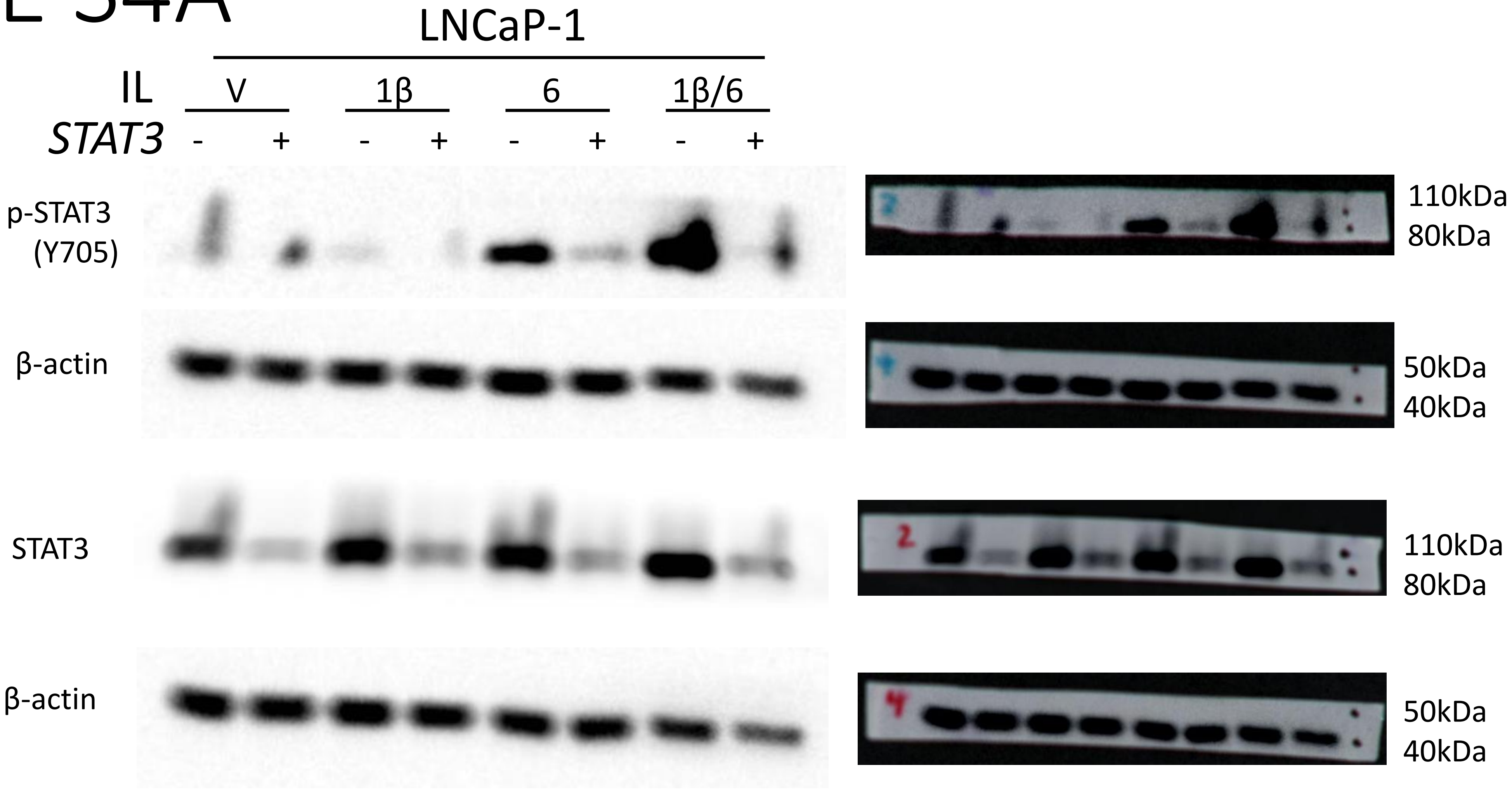
LNCaP-1
IL
V
1β
6
1β/6
STAT3
- + - + - + - +
p-STAT3 (Y705)
β-actin
STAT3
β-actin
110kDa
80kDa
50kDa
40kDa
110kDa
80kDa
50kDa
40kDa

FIGURE S4A

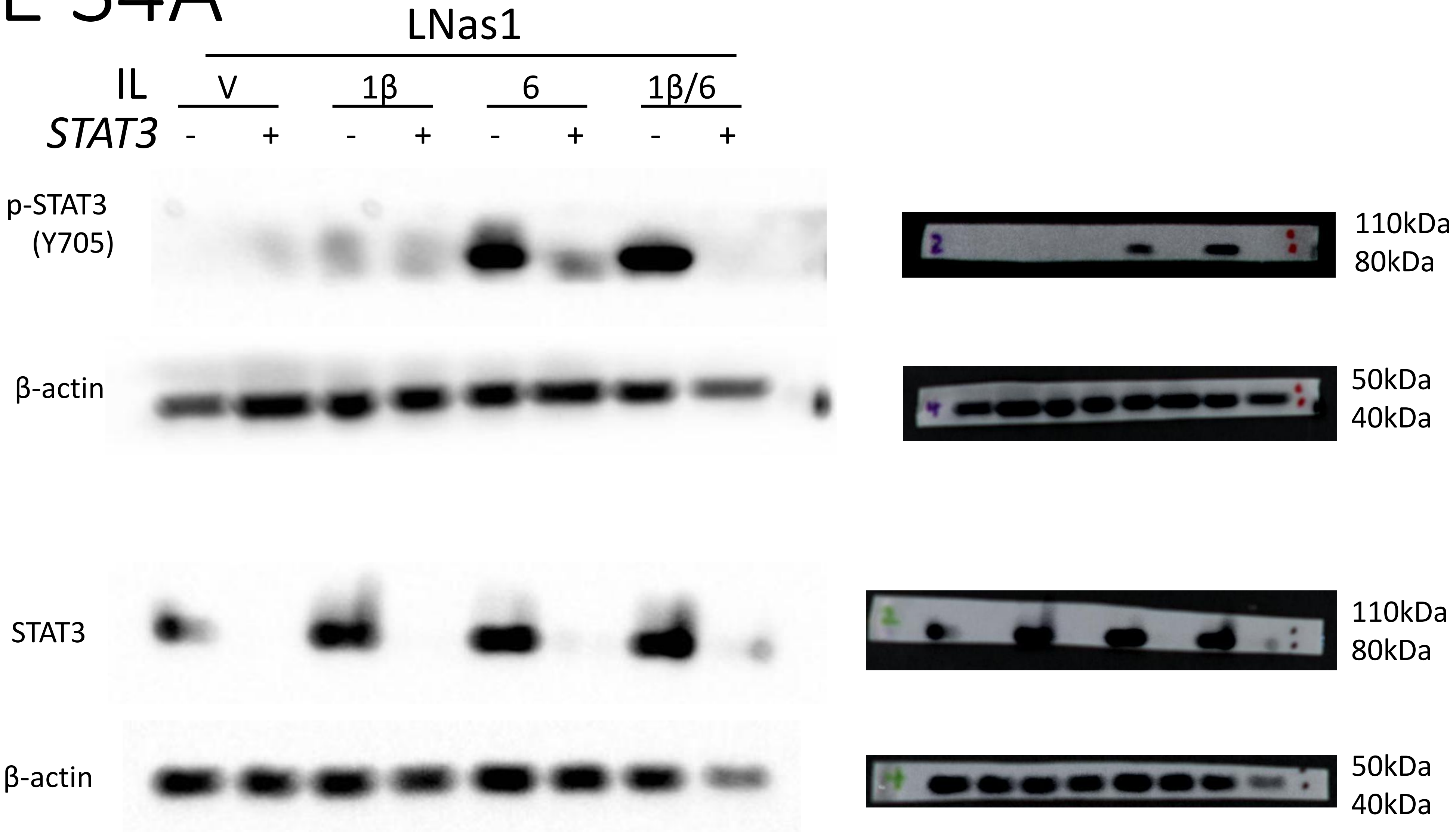
LNas1
IL
V
1β
6
1β/6
STAT3
- + - + - + - +
p-STAT3
(Y705)
β-actin
STAT3
β-actin
110kDa
80kDa
50kDa
40kDa
110kDa
80kDa
50kDa
40kDa

FIGURE S4A

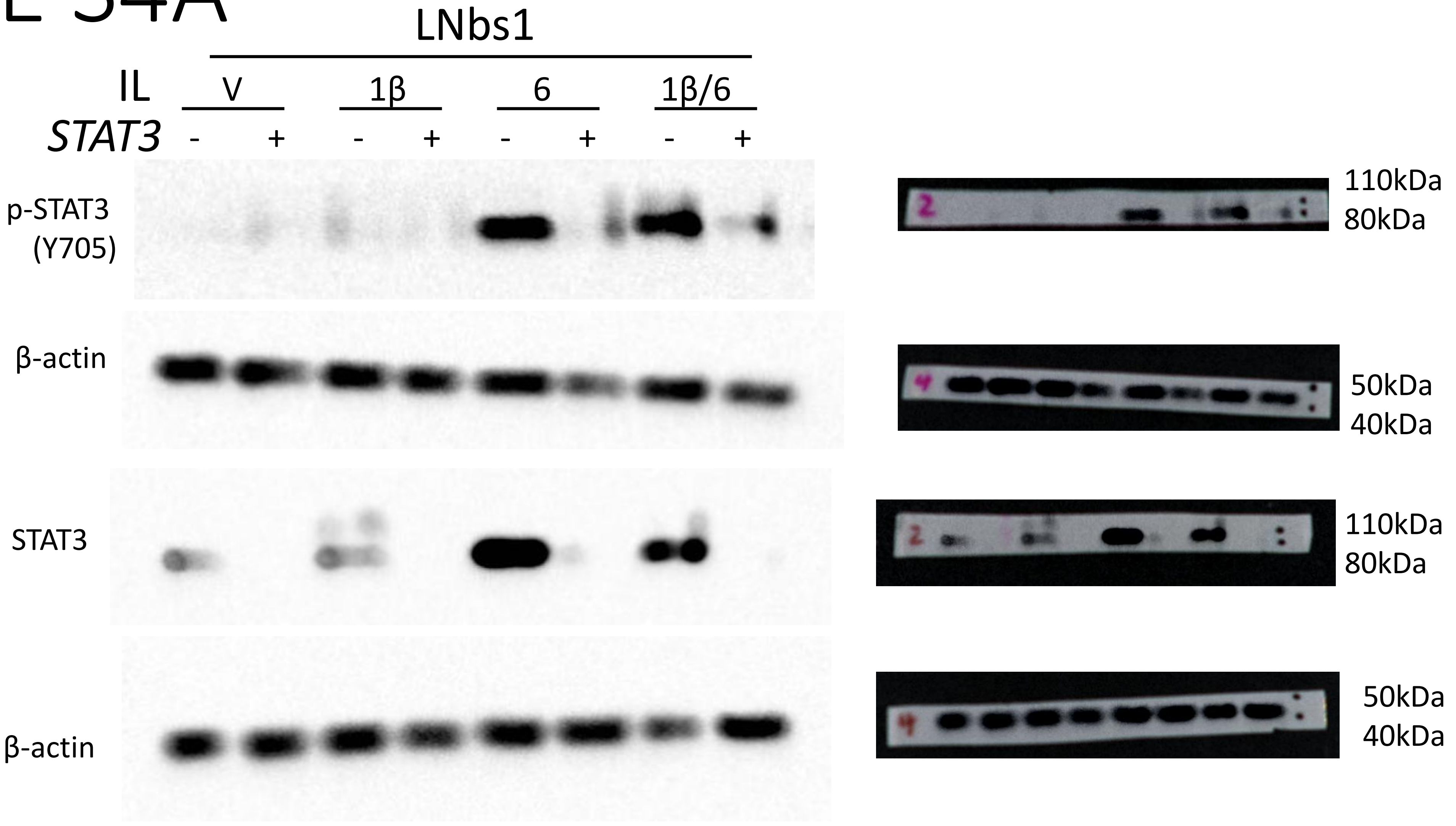
LNbs1
IL
V
1β
6
1β/6
STAT3
-
+
-
+
-
+
-
+
p-STAT3
(Y705)
β-actin
STAT3
β-actin
110kDa
80kDa
50kDa
40kDa
110kDa
80kDa
50kDa
40kDa

FIGURE S4A

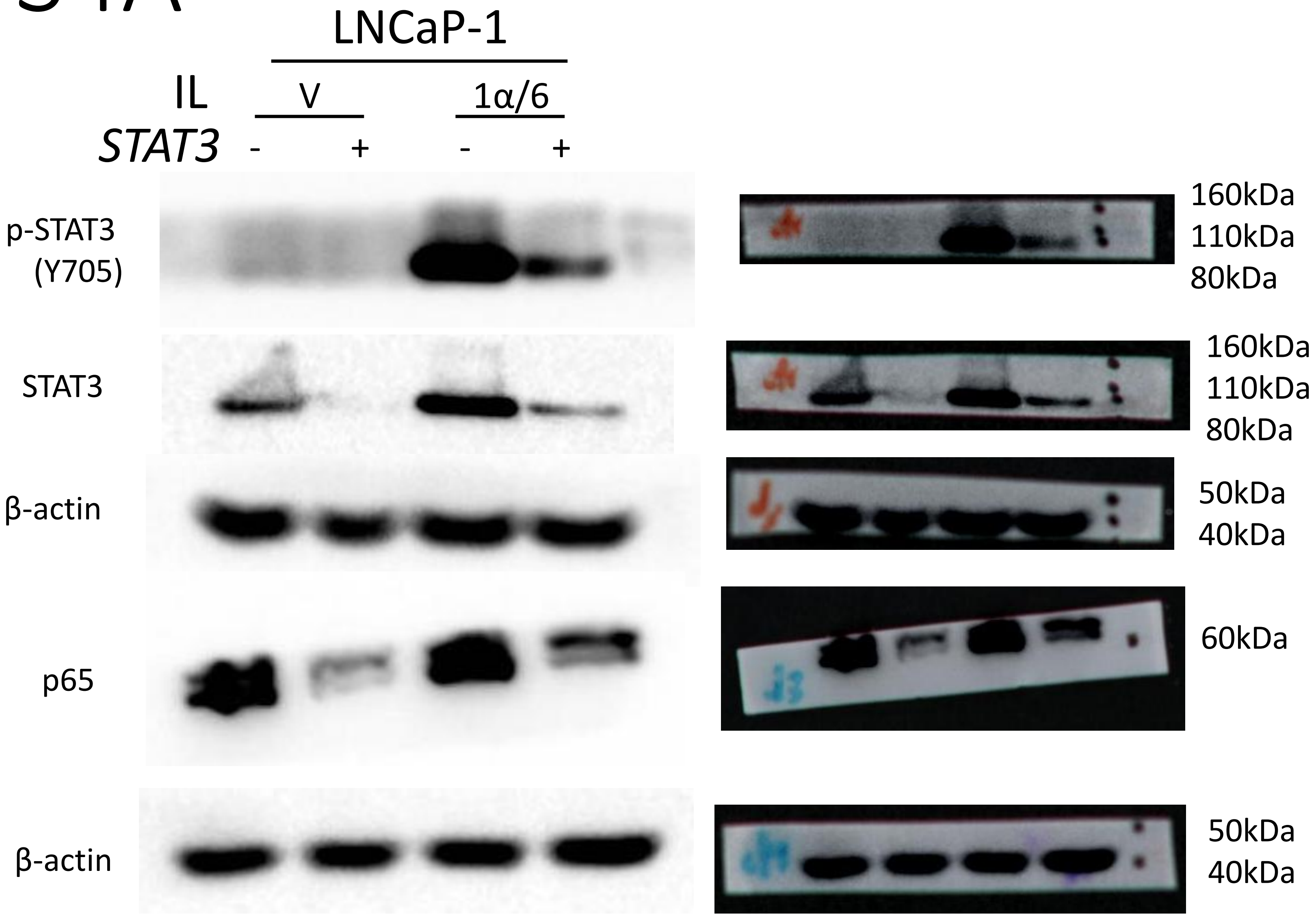
LNCaP-1
IL
V
1α/6
STAT3
-
+
-
+
p-STAT3
(Y705)
STAT3
β-actin
p65
β-actin
160kDa
110kDa
80kDa
160kDa
110kDa
80kDa
50kDa
40kDa
60kDa
50kDa
40kDa
