# Supplementary material for: Chronic IL-1 Exposure Attenuates RELA- and STAT3-Dependent Synergistic Cytokine Signaling in Prostate Cancer Cell Lines
Source: Cancers (Basel). 2025 Nov 26;17(23):3778. doi: 10.3390/cancers17233778 (PMC12691196; doi:10.3390/cancers17233778)
Supplement: Supplementary file 1 [file cancers-17-03778-s001.zip › cancers-3991036-supplementary.pdf]

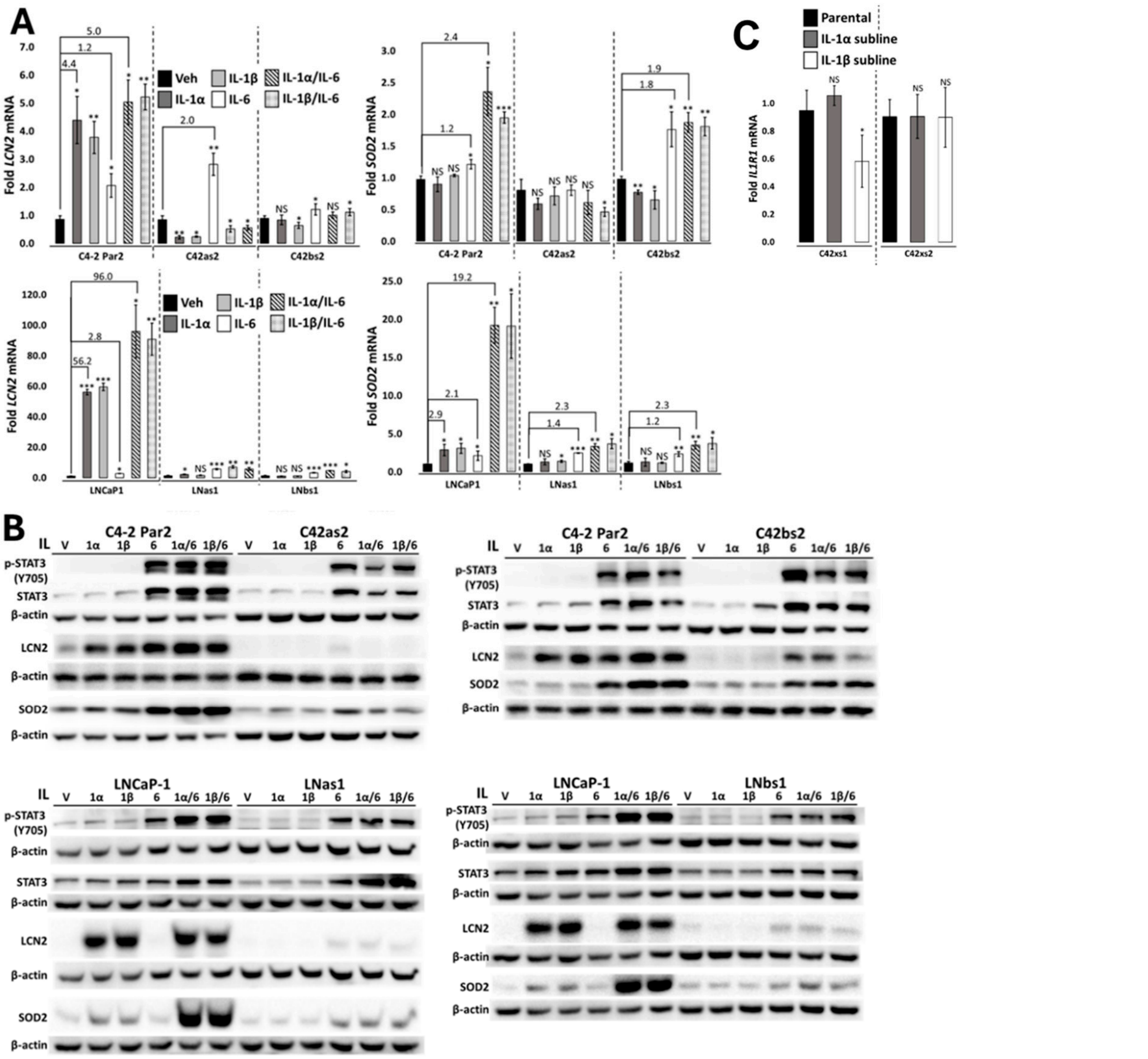

Figure S1. Chronic IL-1 exposure attenuates IL-1 and IL-1/IL-6 intracellular signaling. Parental (C4-2 Par2, LNCaP-1) and chronic IL-1 subline cells (C42as2, C42bs2, LNas1, LNbs1) were treated with vehicle control, 25 ng/mL IL-1α, 25 ng/mL IL-1β, or 100 ng/mL IL-6 alone or in combination acutely for 4 days. (A) RNA and (B) protein were collected and analyzed via RT-qPCR and western blot, respectively. IL-1 sensitivity was assessed using canonical IL-1-induced genes, *LCN2* and *SOD2*. IL-6 sensitivity was assessed using phosphorylated STAT3 (p-STAT3) accumulation. Acute IL-1 exposure induced LCN2 mRNA and protein levels in C4-2 Par2 and LNCaP-1 parental cells, but comparatively, LCN2 induction was not detectable in the C4-2 or LNCaP chronic IL-1 subline cells. IL-1 induced SOD2 in LNCaP-1 cells, but comparatively, SOD2 induction was not detectable in C4-2 Par2 cells or in any of the subline cell lines. IL-6 induced both total STAT3 and p-STAT3 accumulation in the parental and subline cells and induced both SOD2 and LCN2 in the C42bs2 subline, only. Finally, IL-1/IL-6 combination induced enhanced LCN2 and/or SOD2 levels in parental cells, but not in the chronic IL-1 subline cells. (C) RT-qPCR for *IL-1R1* receptor in untreated C4-2 parental and C4-2 subline cells shows that basal *IL-1R1* receptor levels are comparable between parental and subline cells, as previously observed for LNCaP parental and subline cells [12]. Taken together, chronic IL-1 exposure attenuates IL-1 and IL-1/IL-6 intracellular signaling, and this attenuation is not due to reduced receptor levels. *n* = 3 biological replicates; error bars = +/−STDEV; p-value = * ≤ 0.05, ** ≤ 0.005, ***≤ 0.0005. Fold mRNA levels were normalized to vehicle control within each cell line and the numerical values indicate fold change.

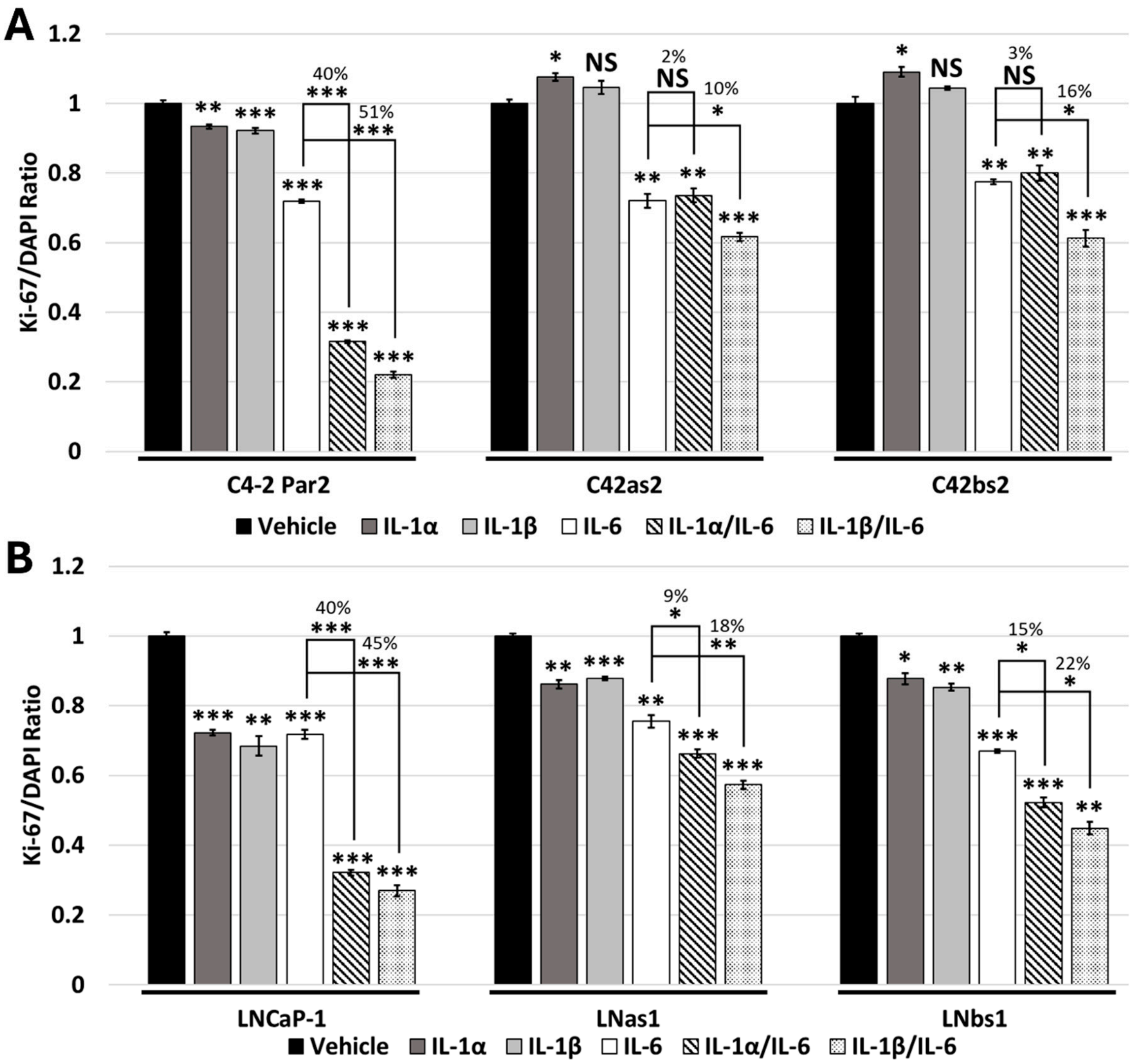

Figure S2. Chronic IL-1 exposure attenuates IL-1/IL-6-induced cytostasis. Parental (C4-2 Par2, LNCaP-1) and chronic IL-1 subline cells (C42as2, C42bs2, LNas1, LNbs1) were treated with vehicle control, 25 ng/mL IL-1α, 25 ng/mL IL-1β, or 100 ng/mL IL-6 alone or in combination acutely for 4 days. Following treatment, cells were immunostained for the proliferation marker, Ki-67, and stained for total cell number with DAPI nuclear stain. The ratio of proliferating to total cells (Ki-67/DAPI) was determined for (A) C4-2 and (B) LNCaP parental and subline cells. (A) IL-6, but not IL-1, is cytostatic for the C4-2 Par2 parental and C42as2 and C42bs2 subline cells. IL-1 and IL-6 in combination enhance cytostasis in C4-2 Par2 parental cells, which is greatly attenuated or lost in the C42as2 and C42bs2 sublines cells. (B) IL-1 and IL-6 alone are each cytostatic for LNCaP-1 parental and LNas1 and LNbs1 subline cells. IL-1 and IL-6 in combination enhance cytostasis in LNCaP-1 parental cells, which is greatly attenuated in the LNas1 and LNbs1 sublines cells. Thus, the chronic IL-1 exposure attenuates IL-1/IL-6-induced cytostasis. $n = 3$ biological replicates; error bars = +/−STDEV; p-value = * ≤ 0.05, ** ≤ 0.005, ***≤ 0.0005. Ratios were normalized to vehicle control within each cell line and numerical values indicate percentage change in proliferation.

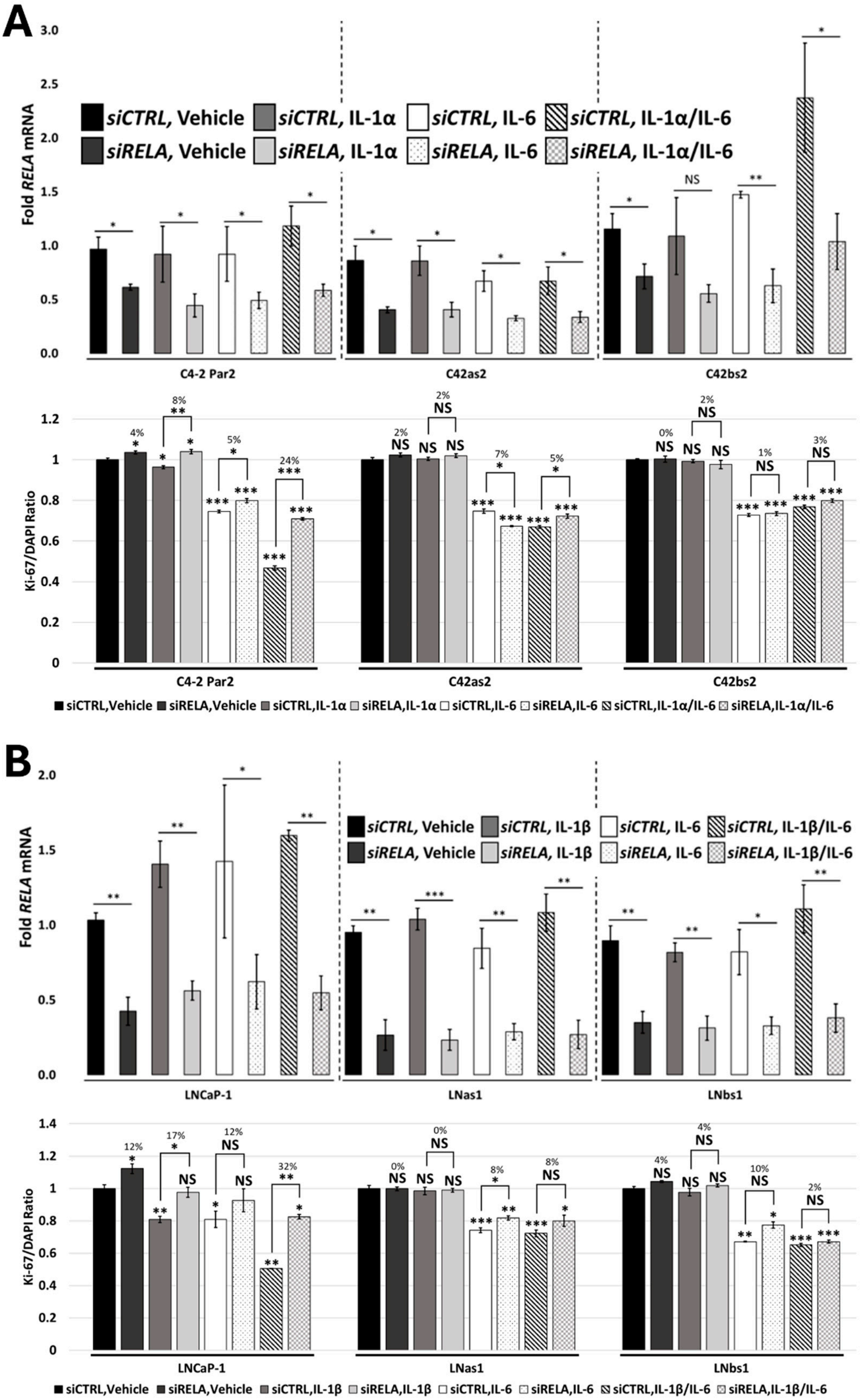

Figure S3. RELA mediates IL-1/IL-6 cytostasis. Parental (C4-2 Par2, LNCaP-1) and chronic IL-1 subline cells (C42as2, C42bs2, LNas1, LNbs1) were transfected with 70 nM control or *RELA* siRNA 1 day before treatment with vehicle control, 25 ng/mL IL-1α, 25 ng/mL IL-1β, or 100 ng/mL IL-6 alone or in combination for 3 days. *RELA* silencing was assessed via RT-qPCR and cells were co-stained for Ki-67 and DAPI to determine the ratio of proliferating cells to total cells for (A) C4-2 and (B) LNCaP parental and subline cells. *RELA* silencing attenuated IL-1 and/or IL-6 cytostasis in parental cells, and to a greater extent for cells treated with the IL-1/IL-6 combination. *RELA* silencing showed no cytostatic rescue in the subline cells. Taken together, RELA mediates IL-1/IL6 cytostasis in C4-2 and

LNCaP parental cells, but not in the subline cells. n = 3 biological replicates; error bars = +/−STDEV; p-value = * ≤ 0.05, ** ≤ 0.005, ***≤ 0.0005. Fold mRNA levels were normalized within the cell line to the vehicle. Ki-67/DAPI ratios were normalized to vehicle control within each subline and numerical values indicate percentage change in proliferation.

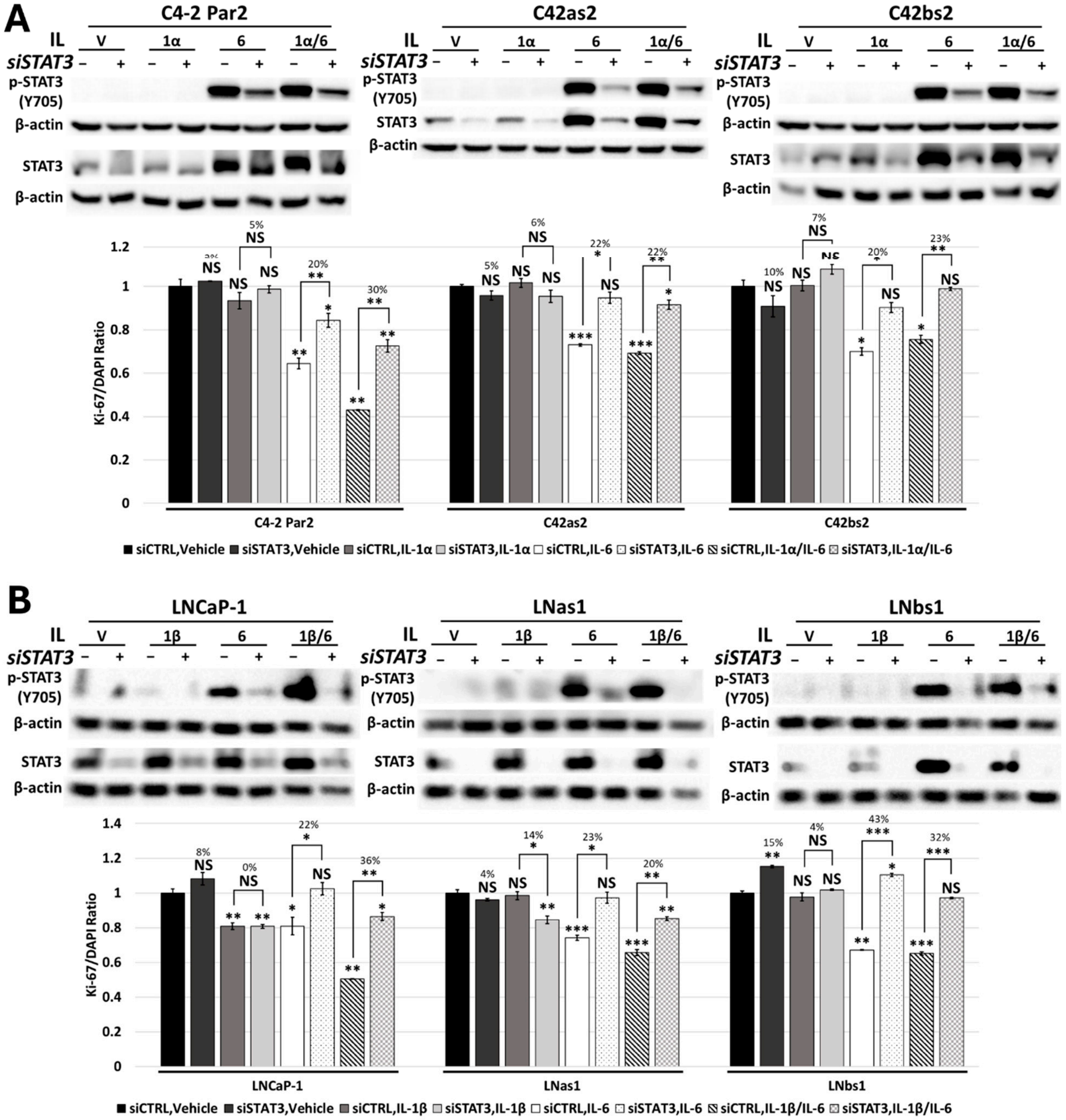

Figure S4. STAT3 mediates IL-1/IL-6 cytostasis. Parental (C4-2 Par2, LNCaP-1) and chronic IL-1 subline cells (C42as2, C42bs2, LNas1, LNbs1) were transfected with 90 nM control or *STAT3* siRNA 1 day before treatment with vehicle control, 25 ng/mL IL-1α, 25 ng/mL IL-1β, or 100 ng/mL IL-6 alone or in combination for 3 days. *STAT3* silencing was assessed via western blot and cells were co-stained for Ki-67 and DAPI to determine the ratio of proliferating cells to total cells for (A) C4-2 and (B) LNCaP parental and subline cells. *STAT3* silencing attenuated IL-6- and IL-1/IL-6-induced cytostasis in parental and subline cells. Taken together, STAT3 mediates IL-1/IL-6 cytostasis in C4-2 parental and subline cells. n = 3 biological replicates; error bars = +/−STDEV; p-value = * ≤ 0.05, ** ≤ 0.005, ***≤ 0.0005. Ki-67/DAPI ratios were normalized to vehicle control within each subline and numerical values indicate percentage change in proliferation.

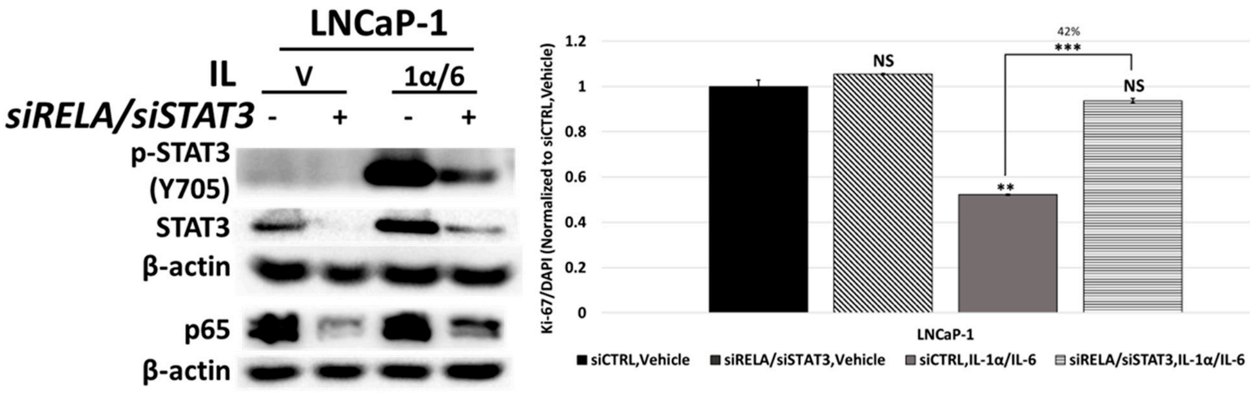

Figure S5. RELA and STAT3 mediate IL-1/IL-6 cytostasis. We transfected LNCaP-1 parental cells with 140 nM control siRNA or 70 nM *RELA* siRNA plus 70 nM *STAT3* siRNA 1 day before treatment with vehicle control or 25 ng/mL IL-1α plus 100 ng/mL IL-6 for 3 days. *RELA* and *STAT3* silencing were assessed via western blot and cells were co-stained for Ki-67 and DAPI to determine the ratio of proliferating cells to total cells. *RELA*/*STAT3* silencing attenuated IL-1/IL-6-induced cytostasis in LNCaP-1 cells, indicating that RELA and STAT3 mediate IL-1/IL-6-induced cytostasis. n = 3 biological replicates; error bars = +/−STDEV; p-value = * ≤ 0.05, ** ≤ 0.005, ***≤ 0.0005. Ki-67/DAPI ratios were normalized to vehicle control and numerical values indicate percentage change in proliferation.
